# Systematic exploration of predicted quaternary structures within pandemic-relevant viral proteomes

**DOI:** 10.64898/2026.09.29.755321

**Published:** 2026-10-01

**Authors:** Yewon Han, Riya Narain, Razan Abbara, Marcelo Querino Lima Afonso, Jeannie Austin, Damian Bertoni, Sooyoung Cha, Alexander I. Cowen-Rivers, Joseph Ellaway, Davide Fransos, Kyle Gion, Darren Hsu, Chantal Hulo, Rachel Seongeun Kim, Oleg Kovalevskiy, Agata Laydon, Jack Mason, Sreenath Nair, Urmila Paramval, Nilkanth Patel, Risha Patel, Ivanna Pidruchna, Jeremy Ratcliff, Maxim Tsenkov, Niccolò Alberto Elia Venanzi, Melanie Vollmar, Newton Wahome, Elisa L.H. Wong, Jennifer Fleming, Sameer Velankar, Milot Mirdita, Philippe Le Mercier, Martin Steinegger, Christian Dallago, Joe Grove

**Affiliations:** 1 School of Biological Sciences, Seoul National University, Seoul, Republic of Korea.; 2 Interdisciplinary Program in Bioinformatics, Seoul National University, Seoul, Republic of Korea.; 3 NVIDIA, Santa Clara, CA, USA.; 4 Stanford University, CA, USA.; 5 PDBe, European Molecular Biology Laboratory, European Bioinformatics Institute, Hinxton, UK.; 6 Google DeepMind, London, UK.; 7 Coalition for Epidemic Preparedness Innovations, Oslo, Norway, London, UK, and Washington, DC, USA.; 8 Department of Precision Medicine, Sungkyunkwan University School of Medicine, Suwon, Republic of Korea.; 9 Biomedical Institute for Convergence at SKKU, Sungkyunkwan University, Suwon, Republic of Korea.; 10 Swiss-Prot group, SIB Swiss Institute of Bioinformatics, Faculty of Medicine, Geneva, Switzerland.; 11 Institute of Molecular Biology and Genetics, Seoul National University, Seoul, Republic of Korea.; 12 Artificial Intelligence Institute, Seoul National University, Seoul, Republic of Korea.; 13 Department of Biostatistics and Bioinformatics, Duke University, Durham, NC, USA.; 14 Department of Cell Biology, Duke University, Durham, NC, USA.; 15 MRC-University of Glasgow Centre for Virus Research, UK.

## Abstract

Mechanistic understanding of viral protein-protein interactions enables global health security and pandemic preparedness, yet experimental characterisation remains difficult, costly, and often restricted to specialised laboratories. Here, we describe an *in silico* campaign using AlphaFold2 and AlphaFold-Multimer to predict monomers and dimer structures within 2,812 viral proteomes from 23 viral families relevant to human health. We report high-confidence predicted structures of 5,279 hetero-, and 2,749 homo-dimers. Structural clustering of interfaces reduces the set fivefold to 1,598 consolidated by form and function, of which 471 (29.5%) lacked detectable similarity to experimentally determined interfaces in the PDB. These clusters yielded two notable findings: new data on vaccinology-relevant glycoproteins, and accurate prediction of the specificity and cleavage-site recognition of viral proteases, which are targets for anti-viral development. Viral monomers and high-confidence dimers are available for interactive browsing in the Pandemic Preparedness Portal of the AlphaFold Database (https://alphafold.ebi.ac.uk/), and the full dataset is available for bulk download.

## Introduction

As exemplified by the COVID-19 pandemic (N. Zhu et al. 2020) viruses pose a persistent threat to global health and security. Mechanistic understanding of their biology can inform the development of interventions such as vaccines and therapeutics, mitigating pandemic risks (Wrapp et al. 2020). With small genomes, viruses rely on genomic economy and protein multi-functionality to ensure a high density of function despite a highly constrained coding capacity (Firth 2014). A common strategy for exerting and regulating function is multimeric self-assembly, driving the formation of both symmetrical structures - such as capsids, replication complexes, or glycoprotein spikes - and asymmetrical assemblies. Knowledge of the structure–function relationships of these complexes guides vaccinology, through rational immunogen design (McLellan et al. 2013) and structure-informed therapeutic development (Faysal et al. 2024).

Thus far, much of our understanding of protein complexes in virology has been provided through experimental structural biology and biochemical assays of direct interaction (Shah et al. 2022). The low throughput of these analyses has limited the scope of investigation to a relatively small number of tractable laboratory strains from priority viruses. Moreover, some viruses have proven recalcitrant to cell culture investigation (Kaur et al. 2026) or require biosafety controls that limit the scale of characterisation.

Existing knowledge of viral protein-protein interactions (PPIs) is distributed across several resources, though most focus on host–virus interactions rather than interactions between viral proteins themselves - for example, VirHostNet, the Host-Pathogen Interaction Database, and P-HIPSTer (Guirimand et al. 2015; Urban et al. 2025; Lasso et al. 2019). Resources describing intra-viral interactions are comparatively scarce, and include databases such as IntAct, BioGRID, the Protein Data Bank (PDB), Swiss-Prot and STRING, which provide some experimentally derived interaction data (Hermjakob et al. 2004; Stark et al. 2006; Szklarczyk et al. 2025; Burley et al. 2019; UniProt Consortium 2025). While these resources offer valuable snapshots of intra-viral interactions, they fall short of the systematic, proteome-wide coverage needed to reveal conserved interaction patterns across viral diversity - a gap that recent advances in structure prediction are positioned to fill.

The advent of highly accurate deep-learning protein structure prediction, for example AlphaFold2 and its multimeric extension, AlphaFold-Multimer (Jumper et al. 2021; Evans et al. 2021), has created the opportunity for *in silico* exploration of PPIs. Exhaustive investigations remain intractable for almost all cellular organisms due to the combinatorial explosion of pairwise searches across a large proteome. Consequently, previous studies have used strategies to reduce the search space; for example, applying classifiers to triage for likely PPIs, drawing on experimental data to select candidates, or pooling multiple protein sequences within each prediction (Humphreys et al. 2021; Han et al. 2026; Todor et al. 2026; J. Zhang et al. 2025; Schmid et al. 2025; Qi et al. 2026). Viruses, however, present an ideal testbed for systematic, at-scale investigation of PPIs, as their compact genomes are computationally tractable for proteome-level, all-vs-all searches. Indeed, this tractability was recently exploited to generate exhaustive AlphaFold-Multimer interactomes for human herpesviruses (T. Zhang et al. 2026; Soh et al. 2024). Extending this approach across a wide range of viral taxa would enable comparative analysis of protein complexes and their interaction modes.

In the present study we use an accelerated implementation AlphaFold-Multimer (Han et al. 2026; Didi et al. 2026) to perform prediction of intra-proteome PPIs across representative viral species from viral families known to infect humans. The rationale for this selection is that recent viral emergence has overwhelmingly occurred within viral families already known to include representatives capable of infecting humans (Woolhouse et al. 2012; J Woolhouse et al. 2013).

Accordingly, this dataset is designed to cover not only known human viruses, but also those with the potential to emerge in the human population. By sampling multiple representatives within each taxonomic group, our approach may uncover shared interactions within or between families or those that define particular genera, allowing explanations of virus biology and evolution based not only on the protein structures they possess (Mifsud et al. 2024) but also the interfaces these proteins create. Common interactions also present attractive targets for the development of broad-spectrum antivirals (Muratore et al. 2012; Link et al. 2020; Stray et al. 2005).

We developed and tested a prediction pipeline against virus-specific benchmark sequences, and then embarked on a campaign of ~1.7M pairwise predictions, across ~42K protein sequences taken from 2,812 viral proteomes. Granular analysis of the frequency of high-confidence dimer predictions across our dataset suggests technical limitations and potential routes to overcoming these, and reveals patterns of protein interaction that correlate with underlying virus biology. Interface-based clustering consolidates our dataset into biologically coherent groups, permitting efficient exploration of form and function. We showcase clusters of vaccinology-relevant viral glycoprotein heterodimers, and complexes that accurately capture the interactions of viral proteases with native substrates. This dataset is made available via the Pandemic Preparedness Portal of the AlphaFold Database (AFDB) (Bertoni et al. 2026), providing new accessible knowledge for hypothesis generation and pandemic preparedness.

## Results

### An exhaustive proteome-level search of viral protein interactions

We selected 2,812 viral proteomes from 23 human-infecting viral families for intra-proteome all-vs.-all pairwise dimer predictions, yielding ~1.7M complex structures containing ~8K high-confidence models (Fig. 1a–c; Methods). In parallel, we also performed structure predictions for the ~42K individual monomers in our sequence set; monomer confidence was highly concordant with Big Fantastic Virus Database (BFVD) v3 ((R. S. Kim et al. 2025; Litvin et al. 2025; Odai et al. 2025; R. S. Kim et al. 2026)) for shared non-polyprotein sequences (Supp. Fig. 1). Notably, this includes mature proteins from polyprotein-encoding viruses (Supp. Fig. 2) for which relatively few structures are available in the AFDB. More than 50K monomeric and high-confidence dimer models are accessible alongside existing viral structures (R. S. Kim et al. 2025; Litvin et al. 2025; Odai et al. 2025; R. S. Kim et al. 2026) via a new Pandemic Preparedness Portal accessible from the homepage of the AFDB (Fig. 1d).

The pool of viral species encompasses a wide diversity of genome compositions, coding strategies and proteome sizes; as a consequence of the combinatorial nature of our approach, DNA viruses with large genomes account for a high proportion of the pairwise predictions (Fig. 1e). For example, the *Poxviridae* contribute ~2% of proteomes but ~60% of all-vs-all pairs. The opposite holds for RNA viruses with small genomes: the *Picornaviridae* account for ~18% of proteomes but ~2% of all-vs-all pairs.

### Benchmark-based validation on the viral dimer prediction pipeline

Decisions about the prediction pipeline were underpinned by a systematic benchmarking against 215 experimental viral complexes deposited in PDB after the training of AlphaFold-Multimer and lacked dimeric structure-level similarity to the pre-training set (Methods; “*positive post-training benchmark set*”). Following Han et al. 2026, we considered a prediction successful if it had ipSAE_max_ ≥ 0.60 and pDockQ2_max_ ≥ 0.23 (Han et al. 2026; Dunbrack 2025; W. Zhu et al. 2023). For pipeline optimization, we compared prediction configurations by recall on the positive benchmark set at the established high-confidence thresholds, whereas the thresholds themselves were selected based on precision and evaluated separately using positive and negative benchmark pairs.

A common limitation when performing protein structure prediction across viral diversity is shallow multiple sequence alignments (MSAs) (Mifsud et al. 2024; Lytras et al. 2026). Recent work shows this can be mitigated through MSA enrichment using the Logan SRA-wide genome assembly (R. S. Kim et al. 2025; Chikhi et al. 2025). Pairwise concatenation of Logan-enriched MSA blocks (R. S. Kim et al. 2026) increased high-confidence yield from 28 to 31 out of 214, and improved ipSAE and pDockQ2 scores (Wilcoxon signed-rank test, P = 0.013; Fig. 2a, Supp. Fig. 3a). Meanwhile, adding filtered paired MSA to unpaired MSAs yielded 32 rather than 31 high-confidence predictions across 214 evaluable pairs, with no significant difference in ipSAE or pDockQ2 scores (Wilcoxon signed-rank test, P = 0.416; Fig. 2b, Supp. Fig. 3b).

Beyond MSA configuration, we studied the effect of the number of AlphaFold-Multimer model weights sampled per prediction. Selecting the highest ipSAE prediction among one, three or five weight sets, resulted in 31, 35 and 40 high-confidence predictions, respectively. Runs with three or five weight sets added 2.1-fold or 2.8-fold GPU cost, and 1.1-fold or 1.3-fold high-confidence predictions compared to single-weight runs (Fig. 2c). High-confidence classification was also robust to recycling depth, with nearly unchanged yields across 4–20 recycles (Supp. Fig. 4).

Based on these results, we chose unpaired Logan-enriched MSAs and single-weight 12-recycle runs as our pipeline configuration. To evaluate the cutoff for the high-confidence predictions, we assembled a benchmark comprising positive post-training benchmark set together with structurally non-proximal experimental negative pairs (Supp. Fig. 5a). Applying the joint high-confidence criterion to the resulting predictions retained 31 of 215 experimentally supported positive pairs, and zero of 221 structurally non-proximal negative pairs, yielding no false-positives at a 14.4% recall (Supp. Fig. 5b). Recall falls below a recent AFDB complex benchmark (~24%) (Han et al. 2026), which exclusively used dimeric assemblies, whereas in this study, we included dimeric chains extracted from larger heteromeric assemblies to increase sample size. This difference in benchmark composition, together with the inherent difficulty of structure prediction for some viral proteins (Lytras et al. 2026), may partly explain the lower recall.

### Patterns of high-confidence interactions across viral proteomes

We observe high-confidence fractions vary with dimer type and protein composition. Of 1,699,311 viral dimer predictions, 8,028 (0.47%) passed the joint confidence filter (Fig. 1b). Despite the recall rate (~14%; Supp. Fig. 5b), this low high-confidence fraction reflects the exhaustive nature of the heterodimer campaign. We took an all-vs.-all approach in an attempt to uncover novel interacting proteins beyond annotations from existing databases, consequently heterodimers constituted the majority of the prediction candidates (97.6% of 1,699,311). Nonetheless, despite the vastly larger number of heterodimer candidates, homodimers constituted a substantial fraction of the high-confidence interactions recovered (34%; Supp. Fig. 7), consistent with the widespread use of homomeric self-association in viral protein complexes (Jayaraman et al. 2016).

In line with our exhaustive approach, heterodimer campaign yielded 0.32% high-confidence predictions, compared to 1% from our previous screen which filtered for dimers in STRING (Han et al. 2026). However, high-confidence yield was lower also for homodimer predictions (6.75% vs. 9.1% from our previous study (Han et al. 2026); Supp. Fig. 6), suggesting differences in protein composition or prediction difficulty for viral proteomes.

A greater high-confidence fraction for homodimers over heterodimers was observed across the majority of viral families with a few notable exceptions, for example, the *Toga-* and *Picornaviridae* (Fig. 3a). In these cases, the frequency of high-confidence heterodimers is increased by consistent prediction of glycoprotein assemblies (e.g. E1/E2, E2/E3) and components of the replication complex (nsp2/nsp4) in the *Togaviridae,* and capsid subunits (VP1/VP2, VP2/VP3) in the *Picornaviridae*.

Capsid proteins form contiguous protein shells and are a major structural component of non-enveloped viruses and many enveloped viruses. Capsid assembly is driven by repeating homo- and hetero-interactions that propagate across symmetry-constrained geometries, and is amenable to perturbation by anti-viral assembly inhibitors (Zlotnick and Mukhopadhyay 2011). Capsids, therefore, represent a paradigm of protein self-assembly - frequently forming both homo- and heteromeric configurations - and provide a useful reference point for our dataset. Unexpectedly, capsid homodimers had a lower high-confidence fraction than other–other (i.e. non-capsid) homodimers (3.96% versus 7.73%). By contrast, among heterodimers, capsid–capsid protein pairs showed an 18.2-fold greater high-confidence fraction than other–other pairs (6.53% versus 0.36%; Fig. 3b,c). This demonstrates that whilst our dataset recapitulates self-assembly of some capsid proteins, including diverse viruses that are poorly represented in the PDB (Supp. Fig. 8), we are also likely to miss many capsid interactions, particularly homomeric ones. This may partly reflect the general difficulty of predicting protein-protein interactions, but capsids present an additional challenge: symmetry-related tiling means that the same capsid protein can participate in several geometrically distinct homomeric interfaces within the assembled shell (Rojas Labra et al. 2026). This is unlikely to be true for most non-capsid homomers in our dataset, and may create ambiguity when modelling simple dimers. Exploring higher-order stoichiometries may therefore rescue some of these missed homo-assemblies.

Polyprotein-derived homodimers similarly had a lower high-confidence fraction than non-polyprotein homodimers (2.21% versus 7.78%; Fig. 3d). As with capsids, this may partly reflect the failure to capture the relevant higher-order stoichiometries in assemblies commonly found in polyprotein-encoding viruses: capsids, glycoprotein spikes and lattices, and replication complexes, for example (den Boon et al. 2024; Haas et al. 2025).

Although many viruses (e.g., picornaviruses) encode a single polyprotein, some encode multiple distinct polyproteins across separate open reading frames or genome segments. We therefore asked whether high-confidence dimer frequency differed within and between polyproteins encoded by the same virus. Heterodimers comprising distinct mature products of the same polyprotein (i.e., intra-polyprotein interactions) reached 3.56% (1,330/37,359) high confidence, compared with no pairs (0/730) from inter-polyprotein interactions (Fig. 3e). The baseline for heterodimers not derived from polyproteins was 0.24%. This enrichment is consistent with polyproteins genetically consolidating functionally related proteins that evolved to act in concert. One clear example from our dataset is the structural polyprotein of the *Togaviridae,* which contains the self-assembling elements of both the capsid and the glycoprotein complex that together form the virion (Chen et al. 2018); we obtained 52 high-confidence complexes of structural proteins from 34 diverse togaviruses.

### Interface-clustering identifies functionally coherent groups of complexes

Searching for interaction interfaces that are conserved across viruses provides a complementary perspective on our dataset, grouping complexes by mode of interaction, rather than purely by composition or genetic relatedness. Following the approach of Strom et al. (Strom et al. 2026), we applied a stepwise clustering strategy (Methods); Foldseek-Multimer (W. Kim et al. 2025) clustering reduced 8,028 high-confidence complexes to 4,951 clusters (a 1.6-fold reduction), while subsequent Foldseek-Interface (Strom et al. 2026) clustering of their representatives yielded a final set of 1,598 interface clusters, a further 3.1-fold reduction and a 5.0-fold reduction overall (Fig. 4a).

This effectively consolidated complexes with shared interfaces, often reflecting a common function irrespective of sequence similarity or even protein composition. For example, the largest cluster (n=391) contained capsid VP2/VP3 heteromers from across the diverse *Picornaviridae*. Foldseek-Multimer initially separated these complexes into 95 clusters, largely correlated with viral genera, whereas interface clustering consolidated them into a single group.

Interfaces conserved across multiple viruses are natural candidates for core, functionally important contacts. Moreover, the structural diversity within individual clusters provides additional insight into how these interactions vary across related viruses. Most were conserved at the family level, consistent with viruses within a taxonomic group sharing fundamental features of virion architecture and replication. Such conserved interfaces may therefore represent attractive targets for broad-spectrum countermeasures. Only seven clusters contained similar dimers spanning multiple viral families. These were predominantly homodimers, from DNA viruses with cellular homologs, consistent with acquisition through horizontal gene transfer. For example, one dUTPase homodimer cluster (n=22) contained proteins of shared origins in cellular hosts (Baldo and McClure 1999; J. Li et al. 2024), from the *Adenoviridae*, *Orthoherpesviridae* and *Poxviridae* (Supp. Fig. 9).

### Clusters of vaccinology-relevant glycoprotein heterodimers

Viral structural proteins assemble into higher-order complexes that form virus particles, enabling their release from infected cells, transport to new host cells, and delivery of the viral genome upon entry (Prasad and Schmid 2012). These proteins frequently act through homo- or heteromeric assemblies and are major targets for adaptive immunity. Structural understanding of such complexes has underpinned rational design of vaccines to maximise protective responses, exemplified by the introduction of stabilising proline substitutions into class-I fusion proteins from RSV, SARS-CoV-2 and HIV (Sanders and Moore 2021). Accurate prediction of the quaternary organisation of viral structural proteins could therefore accelerate structure-informed vaccinology (McCool et al. 2026). As described above, the largest interface-clusters in our dataset comprise heteromeric complexes of *Picornaviridae* capsid proteins. However, these structures are already well represented in the PDB. To assess whether our dataset could instead recover structurally under-characterised viral surface complexes, we turned to viral entry glycoproteins.

Whilst many glycoproteins act as homotrimers (which are absent from our current analyses), our approach captured mechanistically relevant heterodimers, including components of the poxvirus membrane fusion machinery. The *Poxviridae*, particularly Mpox virus, pose an ongoing public-health threat, and have prompted renewed interest in vaccine development (Yu et al. 2025). Poxviruses possess a large entry fusion complex (EFC), containing at least 11 proteins, whose structure and mechanism of action remain incompletely understood. However, recent structural and immunological characterisation of the EFC provides valuable context for assessing our predictions (Lin et al. 2026; Meola et al. 2025; Yang et al. 2023).

Two large interface clusters contain the A16/G9 (n=39) and A28/H2 (n=47) heterodimers, which occupy the central and adaptor layers of the EFC, respectively (Fig. 4b) (Lin et al. 2026). Comparison with experimental structures deposited after the AlphaFold-Multimer training cut-off demonstrates accurate prediction of the heterodimeric arrangement of these mechanistically important proteins (Supp. Fig. 10).

A16/G9 is also an important target of the immune response: immunisation with the complex elicits protective responses, and monoclonal antibodies targeting it are neutralising—including antibodies that recognise quaternary epitopes formed at A16/G9 interface (Meola et al. 2025). One such epitope, identified in vaccinia virus, is structurally conserved across poxvirus A16/G9 dimers in our dataset (Fig. 4c), including highly divergent homologues such as those from *Molluscum contagiosum* (~40% sequence similarity to vaccinia).

Beyond poxviruses, our high-confidence models provide structures for several glycoprotein heterodimers that are absent or poorly represented in the PDB. These include human pegivirus E1/E2 (Fig. 4d), which may exert a novel and currently uncharacterised membrane fusion mechanism (Mifsud et al. 2024), and human herpesvirus-6 gH/gL (Fig. 4e), which mediates interactions with host receptors (Tang et al. 2013). Together, these examples demonstrate how systematic structure prediction can recover conserved viral surface-protein assemblies of direct relevance to structure-informed vaccinology.

### Accurate prediction of viral protease-substrate engagement

Beyond grouping dimers by shared composition, interface-clusters can identify compositionally diverse groups that share a functional interaction mode. Two large, distinct clusters centred on viral cysteine proteases provide a striking example. Picornavirus 3C is a chymotrypsin-like protease (Seipelt et al. 1999), whereas adenovirus protease (L3/23k, AVP) has a unique fold (McGrath et al. 2003). In each interface-cluster, the protease was engaged with a diverse range of native viral substrates, which were consolidated by their shared mode of interaction rather than by protein composition (Fig. 5).

Many viruses encode dedicated proteases that process viral proteins and polyproteins during replication and maturation, and can additionally target host proteins to manipulate cellular responses (X.-D. Li et al. 2005). These enzymes span several established protease folds and virus-specific architectures and display distinct substrate specificities (Rawlings and Bateman 2019). Their essential roles in replication have made viral proteases important targets for direct-acting antivirals, exemplified by therapies for HIV and hepatitis C virus (Bartenschlager et al. 2013).

Picornavirus 3C protease is principally responsible for processing the viral polyprotein and, because we used mature polyprotein products in our predictions, our dataset contains no intact 3C target sequences. Nonetheless, we obtained an interface-cluster of 3C from diverse members of the *Picornaviridae* engaged with three distinct cleavage products: VPg, 2A, and 2B. In each case, the C-terminal region of the cleavage product adopts a similar β-strand conformation at the entrance to the protease active site, positioning its terminal residue - the residue immediately preceding the cleaved peptide bond - adjacent to the catalytic cysteine (Fig. 5a,b). Therefore, these models effectively recreate the configuration of the protease and its product immediately following proteolytic cleavage.

AVP processes structural precursor proteins during adenovirus particle maturation. Unlike picornavirus 3C, complete native AVP substrates were present in our proteome-level pairings, and interface clustering grouped AVP complexes containing three of these substrates: pVI, pX/Mu and pVII. As observed for 3C, AVP substrates formed an upstream β-strand that positioned the cleavage site adjacent to the catalytic cysteine (Fig. 5c,d), recapitulating the geometry of proteolysis. Notably, pVI also serves as an AVP cofactor through its C-terminal peptide, pVIc, which activates the protease by remodelling the active site (Baniecki et al. 2013); this distinct interaction was also captured in our AVP/pVI models (Fig. 5c). Albeit the AVP-pVIc co-factor complex, in the absence of peptide substrate, was in the PDB at the time of AlphaFold-Multimer training (Ding et al. 1996).

To test whether these observations reflected authentic substrate recognition, we quantified AVP selectivity across the complete dataset, using the many non-substrate pairings as an internal negative control. Only ~1% of AVP interactions with non-substrate proteins reached high confidence, compared with ~40%, ~20% and ~5% for the native substrates pVI, pX/Mu and pVII, respectively (Fig. 5e). Thus, AVP showed substantial discrimination between substrates and non-substrates, although two additional native substrates, pIIIa and pVIII, yielded no high-confidence interactions. Viral proteases must recognise not only the correct substrate proteins but also specific cleavage sites within them. We therefore asked whether our predictions reproduced AVP sequence specificity by aligning substrate sequences according to their three-dimensional proximity to the catalytic site and generating sequence-logo plots alongside matched distance measurements (Fig. 5f). This recovered the key sequence features of a canonical AVP cleavage motif: (M/I/L)XGG–X. Taken together, these data demonstrate accurate and precise modelling of viral protease-substrate interactions.

Previous small-scale investigations have demonstrated that AlphaFold-Multimer can recapitulate interactions of cellular proteases with peptide targets (Zlobin and Golovin 2022; Veizaj et al. 2026; Ge et al. 2024). Here, we extend this principle to unbiased proteome-scale searches across diverse viruses, recovering substrate selection, cleavage-site specificity and mechanistically coherent interfaces. More broadly, the ability to systematically identify conserved molecular mechanisms across viral diversity may provide starting points for the discovery and design of antiviral inhibitors targeting shared viral vulnerabilities.

## Discussion

Building on other community-led efforts (R. S. Kim et al. 2025, 2026; Litvin et al. 2025; Mifsud et al. 2024; Odai et al. 2025; Nomburg et al. 2024; Soh et al. 2024), our study represents an exhaustive search of protein-protein interactions across diverse viral proteomes through AlphaFold-Multimer protein-structure prediction. While the wider virosphere remains a formidable target for systematic investigation, with ~18K viral genomes taxonomically defined by the ICTV, our focus on viral families known to contain human-infecting viruses maximises the potential for hypothesis generation relevant to the development of anti-viral countermeasures. Moreover, computational analyses permit investigation of viruses irrespective of their amenability to growth in the laboratory, challenges of biocontainment, or availability of model systems and tools, thereby enabling exploration across broad viral diversity and potentially revealing targets for broad-spectrum intervention.

Guided by an experimentally grounded benchmark dataset, we employed a prediction pipeline that balanced accuracy, computational efficiency and the scale required for brute-force all-vs.-all searches. Stringent filtering (Han et al. 2026) yielded a high-confidence dataset with a low expected false-positive rate, but consequently a low overall frequency of retained models. While this is unsurprising given the combinatorial scale of our approach, patterns within the dataset also point to specific failure modes. For example: capsid proteins produced fewer high-confidence homomeric predictions than non-capsid proteins, despite their well-established propensity for self-assembly. One explanation is inadequate sampling of oligomeric space, such that some homomeric interactions may only be recovered at higher stoichiometries. The same may apply to viral glycoproteins, many of which form trimers. Exploring protein stoichiometry is therefore likely to be important for accurately modelling the symmetric assemblies that comprise virus particles and are particularly relevant to vaccine development. However, it should be noted that recent work suggests that AlphaFold may itself have limitations in modelling higher-order symmetric assemblies of viral structural proteins (Rojas Labra et al. 2026).

Deploying interface-based clustering (Strom et al. 2026) was highly effective in consolidating functionally related complexes across substantial sequence and structural divergence. This revealed conserved interaction modes shared across viral families and, in the case of proteases, mechanistically related assemblies formed by compositionally distinct proteins. We expect this approach to be particularly useful for navigating large and structurally diverse datasets and demonstrate its utility here by grouping highly diverse poxvirus glycoprotein assemblies.

Recent studies suggest that integrative structure prediction modelling of viral glycoproteins can illuminate interactions with the human immune response (Paciello et al. 2025) and guide immunogen design (McCool et al. 2026). Systematic prediction of glycoprotein complexes, together with other structurally important viral assemblies such as capsids and secreted proteins (Akey et al. 2014), could therefore provide a valuable stockpile of molecular knowledge for both proactive and outbreak-responsive vaccinology.

One capability exposed by our systematic approach is the ability of AlphaFold-Multimer to accurately model the substrate selectivity, sequence specificity and molecular mechanism of cysteine proteases from the *Picornaviridae* and *Adenoviridae*. Understanding whether this capability has arisen *de novo* or represents generalisation from distantly related proteases in the PDB, will require further investigation. Nonetheless, it could have broad applications, from investigating the molecular determinants of substrate specificity in diverse viruses to identifying previously unknown cleavage sites within viral proteins. Given the central role of proteolytic processing in the replication of many viruses, such mechanistic insight could also complement ongoing efforts to develop protease inhibitors as broad-spectrum antivirals (Nelen et al. 2026; Lithgo et al. 2024).

More widely, systematic interrogation of this and future datasets may reveal further unanticipated examples in which AlphaFold-Multimer captures molecular mechanisms or discovers novel interactions that have eluded comparable experimental screens. Our findings illustrate the potential for *in silico* exploration of protein-protein interactions in any viral proteome, revealing and consolidating knowledge of viral mechanisms, while offering a scalable route to rapidly analyse and respond to emerging pathogens.

The AlphaFoldDB Pandemic Preparedness Portal, through which predictions from this and future campaigns will be made available, represents a foundation for systematic exploration of structure, function and mechanism across diverse viruses. This has the potential to enable and accelerate fundamental discovery in molecular virology and, critically, the development of antiviral countermeasures: diagnostics, therapeutics and vaccines.

## Biosecurity statement

A general concern about virus research - whether *in vitro* or *in silico* - is that the practice may lead to manipulations of viruses that result in accidental or intentional increases in transmission or virulence. Cognisant of this, we limited our analysis strictly to interactions within each viral proteome, and chose not to explore interactions across proteomes, or between viral proteins and host proteins. Therefore, we may learn about how viral proteins assemble and, through this, how we might target viruses with drugs or vaccines. We will not learn about interactions between viral and host proteins that drive pathogenicity. The complexes are based on sequence data observed in nature, and we do not explore the impact of non-observed mutations on complex confidence. By making these choices, we aimed to maximise the potential benefits of our work, whilst minimising risks. Given this, the biosecurity risk associated with our dataset is low.

## Methods

### Sequence and prediction candidate selection.

There are 34 families of viruses known to infect humans (De Castro et al. 2024). To find well annotated sequence representatives across these families, we selected the International Committee on Taxonomy of Viruses (ICTV) Virus Metadata Resource (VMR; VMRv40.1) as a definitive and current record of exemplar species. The VMR also maps directly to the viral species included in UniProt, permitting us to derive proteome level sequence sets for each species. Many viruses encode simple open reading frames where one gene produces one protein, in these instances protein sequences can proceed directly to structure prediction. However, 15 of the human infecting families encode one or multiple polyproteins, where one gene produces a long polypeptide that is proteolytically cleaved to produce multiple mature proteins. In these cases accurate polyprotein sequence annotations are essential for biologically meaningful prediction of structure and interaction. The ViralZone team's recent efforts have provided high-quality sequence annotations for four polyprotein encoding families, including the *Picornaviridae* which contains >500 exemplar species (Supp. Fig. 2). These well-annotated viruses were included in the current study, whilst the 11 families (~700 species) without consistent and accurate annotations were excluded (e.g. *Coronaviridae* and *Retroviridae*). This resulted in a final input sequence set of 2,818 proteomes containing a total of 42,180 individual proteins, including 7,703 polyprotein mature products. Notably, VMRv40.1 contains the legacy-definition of the *Flaviviridae* prior to its recent taxonomic reorganisation (Simmonds et al. 2025). In this study we used the previous definition, in which the *Flaviviridae* contains four genera: hepacivirus, orthoflavivirus, pegivirus, and pestivirus. Monomer and homodimer candidates were derived from the 42,180 individual proteins. 1,677,356 heterodimer candidates were derived by pairing all proteins within a viral genome (intra-proteome all-vs-all). No inter-proteome heterodimers, including viral-host interactions were predicted.

### Benchmark dataset construction.

Proximal pairs of chains from complexes of viral proteins were identified as a positive set by combining SIFTS-based PDB-to-UniProt mappings with structural proximity detected using Foldseek createdimerdb. Among 40,918 proximal chain pairs grouped into 5,870 structural clusters, the intersection of 19,780 viral PDB chain pairs mapped to UniProt via SIFTS yielded 17,619 candidate pairs supported by both sources. Structural clustering, redundancy reduction, representative selection, and post-training filtering against the PDB were performed following the previous approach (Humphreys et al. 2021; Han et al. 2026; Todor et al. 2026; J. Zhang et al. 2025). This yielded 2,798 representative PDB chain pairs, which were mapped to 943 unique pairs of UniProt accessions. After removing eight pairs containing host proteins, 935 viral pairs remained (571 bacteriophage, 361 non-phage, and three unclassified). From the 361 non-phage pairs, we retained non-polyprotein pairs with a BFVD v3 Logan-block match (R. S. Kim et al. 2026), yielding 215 positive pairs used in the final benchmark.

Negative pairs were derived from the same PDB structures supporting the 215 positive pairs. These positives mapped to 140 PDB structures containing 1,694 protein chains, from which 25,966 candidate chain pairs were enumerated. Pairs were excluded if they belonged to the same PDB entity, were spatially proximal according to createdimerdb, or the viral PDB interaction set mapped to UniProt via SIFTS, contained obsolete, sequence-unavailable, or manually excluded UniProt accessions, represented the same accession, had PDB-chain coverage < 70% of the corresponding UniProt sequence, or contained a protein ≥ 1,300 amino acids.

Potential near-identical sequence pairs were identified using MMseqs2 at sequence identity ≥ 0.9 and bidirectional coverage ≥ 0.8. Known interacting pairs were filtered using the full 935-pair viral set, the viral PDB interaction set mapped to UniProt via SIFTS, and createdimerdb pairs mapped to UniProt. This filter list was further expanded by substituting either or both proteins in each known interaction with their MMseqs2-defined sequence neighbors, increasing the filter list from 1,564 to 2,049 pairs. After all filters, 1,998 candidate negative pairs remained. Up to five negatives were selected per positive from its source PDB structure(s), prioritizing positives with smaller candidate pools and enforcing global accession-pair deduplication; random selection used seed 42. This yielded 242 unique negative pairs, from which pairs with a BFVD v3 Logan-block match were retained, resulting in 221 negative pairs used in the final benchmark. Negative labels indicate absence of spatial proximity in the observed PDB assemblies rather than absence of interaction in all biological contexts.

A pair was considered evaluable if prediction and all subsequent post-processing and metric evaluation steps were completed successfully. Of the 215 pairs in the positive set, 214 were evaluable for the analyses shown in Fig. 2a,b.

### Multiple Sequence Alignment generation

Multiple Sequence Alignments (MSAs) were generated for all 42,180 unique monomer sequences using colabfold_search (ColabFold 1.6.2) with the MMseqs2-GPU backend (Kallenborn et al. 2025), searching against UniRef30 and the ColabFold metagenomic database (--use-env 1). Dimer MSAs were generated directly in unpaired mode from concatenated two-chain input sequences. For polyprotein-encoding sequences, MSAs were generated directly from the mature product sequences (cleaved segments), as provided in the production FASTA, rather than from full-length polyprotein sequences followed by cropping.

### Structure prediction

Dimer structures were predicted using an accelerated implementation of OpenFold leveraging NVIDIA BioNeMo Inference Runtime with AlphaFold-Multimer v3 weights (model_1_multimer_v3) and 12 recycling iterations. Monomers were predicted using ColabFold v1.6.2 with alphafold2-ptm weights and 12 recycling iterations with early stopping. Failures due to out-of-memory errors or were observed in the presence of large MSAs and long sequences (greater than around 2100 amino acids). These failure modes occurred in 14,994 predictions and were not further investigated. Computation for any one complex was limited to four hours of execution time, and predictions exceeding this limit were not completed and excluded from downstream analysis. Among the 1,719,536 input dimeric pairs, a total of 1,704,542 dimeric structures were predicted, comprising 1,663,511 heterodimers and 41,031 homodimers.

### High-performance-compute scaling

MSA generation and structure prediction were scaled following (Han et al. 2026). Dimer predictions were distributed across NVIDIA DGX H100 and A100 nodes; pairs with combined sequence length ≤900 amino acids were predicted on A100 nodes, while longer pairs on H100 nodes.

### Post-processing toolkit and data products

A prior post-processing pipeline (Han et al. 2026) was extended to correctly propagate polyprotein fragment metadata (including sequence coordinates, protein name, and fragment status) through the chain-binding step, ensuring fragment segments were accurately represented in released data products. Models containing non-standard amino acids or coordinate-sequence mismatches were excluded prior to post-processing. Of 1,704,542 folded dimers, 1,699,311 (1,658,565 heterodimers and 40,746 homodimers) were successfully post-processed, of which 8,028 met the high-confidence threshold of ipSAE_max_ ≥ 0.6 and pDockQ2_max_ ≥ 0.23. Of 42,180 monomers, 41,869 were successfully post-processed. Among the 7,703 polyprotein monomeric entries, 146 predictions failed, leaving 7,557 successfully predicted monomeric structures. No quality thresholds were applied to monomers as ipSAE and pDockQ2 are interface metrics not applicable to single-chain structures.

### Assigning viral families

Every predicted model carries the NCBI taxonomy identifier (taxid) of the virus it came from, and the 1,699,311 models span 2,783 distinct identifiers. Each was resolved to a full lineage against a single fixed release of the NCBI taxonomy (downloaded 2026-09-09). Taxids that NCBI has since renumbered or withdrawn were followed through its merged- and deleted-node lists rather than discarded. 2,782 of the 2,783 taxids resolved to a family directly. The unresolved taxid had been withdrawn from the taxonomy and was assigned to the family shared by all other taxids from the same virus species. Viral identity was confirmed by tracing each lineage to the NCBI “Viruses” root node (taxid: 10239). This result yielded 25 families, which consolidated into 23 when nesting *Hepaciviridae* and *Pestiviridae* within *Flaviridae*, consistent with ICTV release VMR 40.1.

### Protein classification

Each modelled viral protein chain was identified by its UniProt accession and residue range, and classified as capsid, other or unclassified. Capsid-shell and core/nucleocapsid proteins were annotated separately but combined in the capsid category for compositional analyses. Non-polyprotein standalone proteins were classified using, in order of precedence, UniProt capsid keywords, capsid Gene Ontology (GO) cellular-component terms, nucleoprotein/nucleocapsid keywords and GO terms, and protein names. Annotations for capsid assembly or maturation, genome packaging, matrix, tegument and generic ribonucleoprotein complexes were not considered evidence of capsid identity. For name-based assignments, cleavage-product lists were removed from precursor names, and scaffolding and chaperone proteins were excluded from capsid assignment. Reviewed UniProtKB/Swiss-Prot entries lacking capsid or nucleocapsid evidence were assigned to the other category. Meanwhile, mature polyprotein products were only classified by product name rather than precursor-level annotations, using a classification table covering all 77 distinct product names in the dataset (Supp. Table 1). Chains without an assignment, including polyprotein chains spanning multiple mature products, remained unclassified. The supporting evidence was recorded for each chain. Among 41,774 distinct chains from 34,964 UniProt accessions represented across the 1,699,311 predicted dimers, 7,183 were classified as capsid proteins (6,606 capsid and 577 core/nucleocapsid), 31,610 as other, and 2,981 as unclassified. Dimers were classified as capsid–capsid, capsid–other or other–other; dimers containing an unclassified chain were reported separately.

### Interface Clustering of PDB

PDB structures downloaded in January 2026 were processed using foldseek (commit d6204679), following the pipeline and parameters described in the Foldseek-Interface ((Strom et al. 2026)). Dimers extracted using createdimerdb were clustered with easy-multimercluster at TM-score and chain TM-score thresholds of 0.9 and 0.5, respectively. Cluster representatives were compiled into repdb, and interfaces were extracted using createinterfacedb. These interfaces were then clustered with easy-multimercluster using exhaustive search, TM-score and lDDT thresholds of 0.4 and 0.20, respectively, and an E-value cutoff of 10^7^, yielding 118,269 interface clusters.

### Interface clustering and search of the high-confidence subset

The 8,028 high-confidence viral dimers (ipSAE_max_ ≥ 0.6; pDockQ2_max_ ≥ 0.23) were clustered using Foldseek (commit d6204679), following the two-stage pipeline and parameters described by Strom et al. ((Strom et al. 2026)). First, the di-mer clustering step yielded 4,951 clusters (a 1.6-fold reduction), followed by an interface clustering step that yielded 1,598 interface clusters.

Interfaces extracted from all 8,028 high-confidence viral dimers were searched against 118,269 PDB interface cluster representatives. This search followed the HumanPPI-to-PDB procedure described by Strom et al. (Strom et al. 2026), using easy-multimersearch (Kim et al., 2025) with --cov-mode 0 and otherwise default search parameters. A PDB hit was defined as max(qTM, tTM) > 0.4, where qTM and tTM denote query- and target-normalised TM-scores, respectively. A cluster was considered PDB-matched if at least one member had a qualifying hit. Overall, 6,598 dimers (82.2%) had PDB hits, whereas 471 of the 1,598 interface clusters (29.5%) had no PDB hit (Fig. 4a).

## AdV protease/substrate alignment and sequence logo analysis

Substrate sequences were aligned by structure, rather than by sequence, using a single large interface-cluster (n=69) of AdV protease/substrate pairs. For each complex we measured the distance from the catalytic cysteine sulfur to the backbone carbonyl carbon of every substrate residue and took the nearest as an anchor, this being the carbon a cysteine protease attacks. Each substrate sequence was then read as a window of three residues either side of the anchor, and the windows stacked, so every column holds the same offset from the anchor. Because the anchor is measured in each structure independently the resulting sequence motif arises purely from geometric arrangement in the active site. Of the 69 models in the cluster, 65 were used; the remaining four were excluded because no part of the substrate reaches the active site. Per column, Shannon information content was calculated with the standard small-sample correction, and logos were drawn with logomaker (Tareen and Kinney 2020), with letters scaled by frequency and coloured by residue class.

### Molecular modelling

Visualisation of protein structures was performed with Mol* (Sehnal et al. 2021).

## Supplementary Material

1

## Figures and Tables

**Figure 1:**
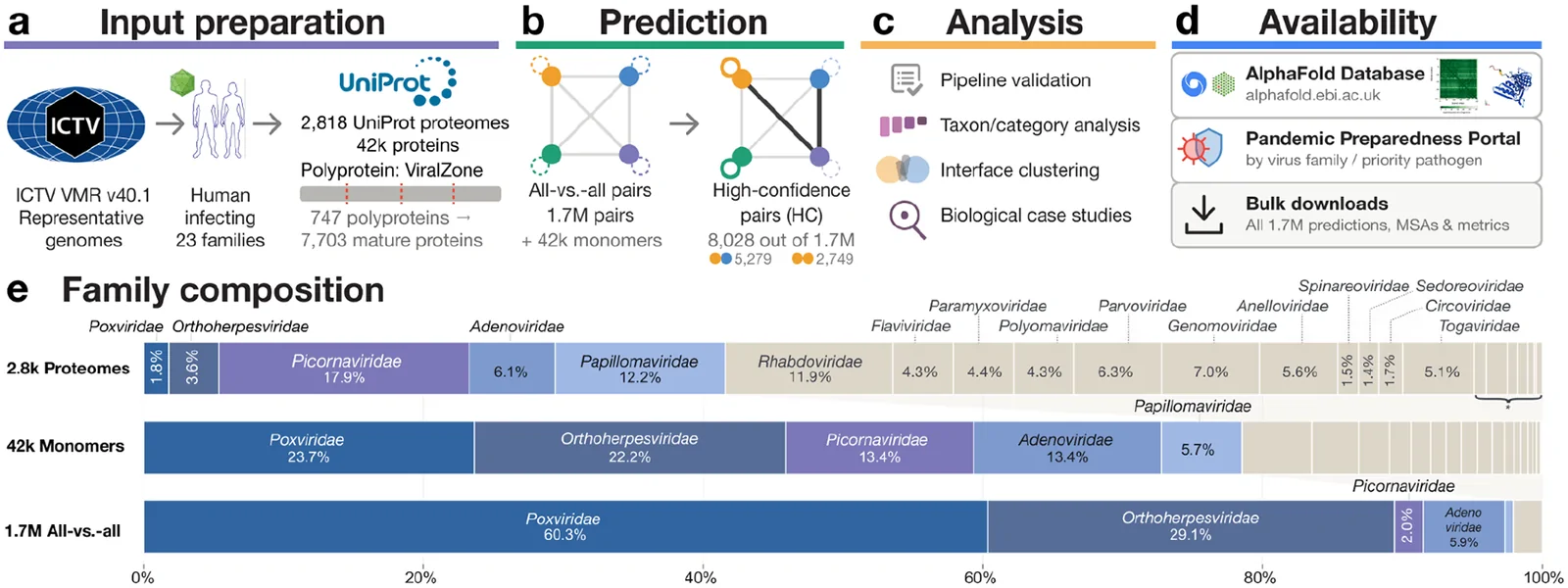
Proteome-wide dimer prediction for human-infecting viruses. **a,** We selected representative genomes of 23 human-infecting virus families from the International Committee on Taxonomy of Viruses Virus Metadata Resource (ICTV VMR) v40.1. **b,** We retrieved 2,818 Proteomes from UniProt and split polyproteins into mature peptides (ViralZone); all intra-proteome pairs were enumerated (1.7M pairs). **c,** The 1.7M dimeric and 42k monomeric structures were predicted (Methods), yielding 2,749 homodimers (6.75%) and 5,279 heterodimers (0.32%), together 8,028 dimers met *high-confidence (*Han et al. 2026*)* and were analysed for biological case studies. **d,** High-confidence dimers are available in the AlphaFold Database; all predictions via bulk download. **e,** Taxonomic breakdown of viral proteomes, monomeric proteins and all-vs-all pairs. Unlabelled segments (*) correspond, from left to right, to: Caliciviridae (0.9%), Hepadnaviridae (1.5%), Orthomyxoviridae (0.7%), Bornaviridae (0.7%) Filoviridae (0.5%), Pneumoviridae (0.1%) and Kolmioviridae (0.4%).

**Figure 2.**
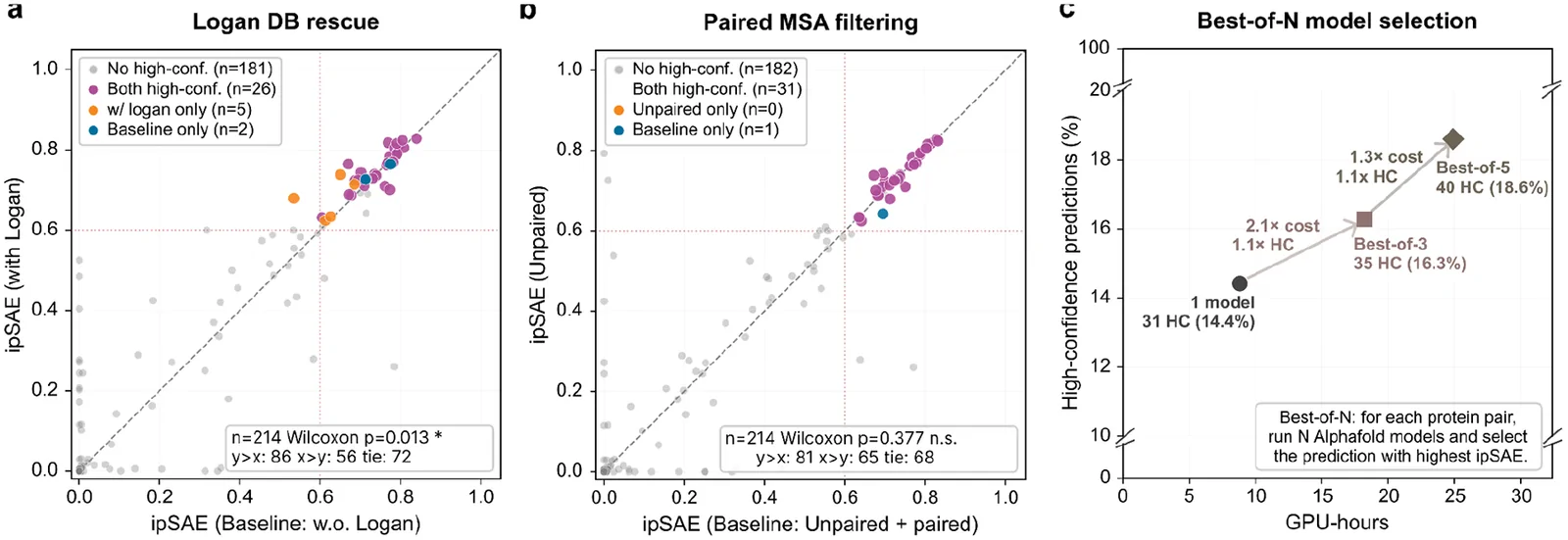
Effects of MSA composition and model selection on prediction yield and computational cost. Analyses used a set of 215 experimental viral complexes, with 214 evaluable pairs in a and b. High-confidence predictions satisfied ipSAE_max_ ≥ 0.60 and pDockQ2_max_ ≥ 0.23. In a and b: the production configuration is plotted on the y-axis; orange is high-confidence with the production configuration only, blue, with the alternative only, purple for both; dotted lines mark ipSAE = 0.6; P-values are from Wilcoxon signed-rank tests comparing paired ipSAE scores. **a,** Logan-block, enriched MSAs, concatenation improves prediction yield. ipSAE scores without (x-axis) and with (y-axis) the Logan block are compared (P = 0.013). Concatenation retained 26 high-confidence predictions, gained five and lost two. **b,** MSA pairing shows no significant change in ipSAE. Scores from paired-plus-unpaired MSAs (x-axis) are compared with those from unpaired-only MSAs (y-axis; P = 0.416). **c,** Best-of-*N* model selection offers modest gains but incurs increased computational cost. Using model 1 alone or selecting the highest-ipSAE prediction from three or five AlphaFold-Multimer models yielded 31, 35, and 40 high-confidence predictions at 1.0×, 2.1×, and 2.8× GPU cost, respectively.

**Figure 3.**
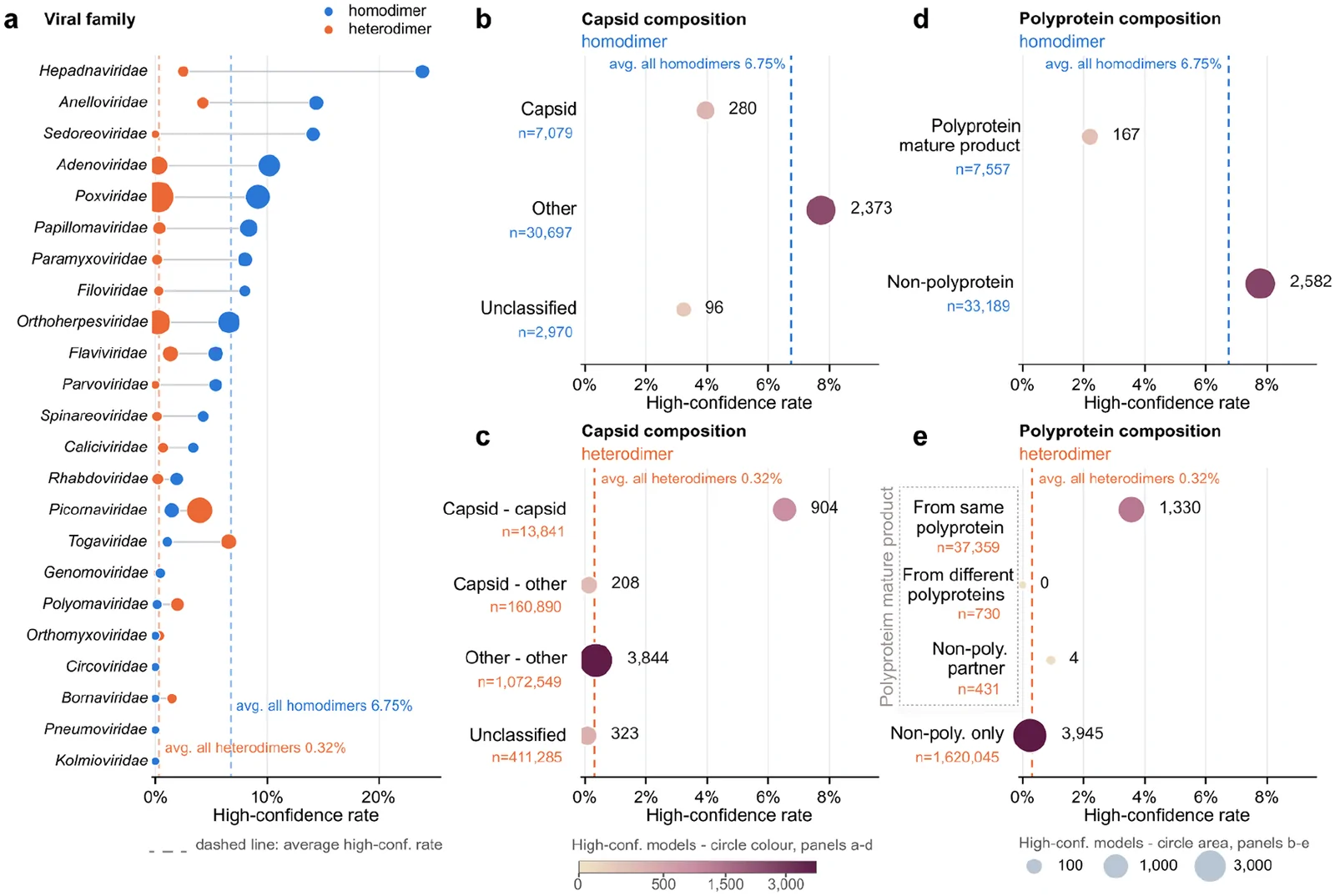
High-confidence fractions across viral dimer types, protein compositions and families. **a,** High-confidence fractions across all 23 NCBI taxonomic families, ordered by homodimer fraction. Connected dots compare dimer types within each family; colour identifies dimer type and circle area encodes high-confidence count as in b–e. In b-e, the dashed lines indicate overall homodimer and heterodimer fractions. **b,c,** High-confidence fractions by capsid composition for homodimers (b) and heterodimers (c), Other indicates non-capsid proteins; unclassified are entries with ambiguous protein identities. **d,e,** High-confidence fractions by polyprotein composition for homodimers (d) and heterodimers (e). In b–e, dot position indicates the high-confidence fraction; circle area and colour encode high-confidence counts using square-root scaling. Adjacent labels give high-confidence counts; n denotes input counts.

**Figure 4.**
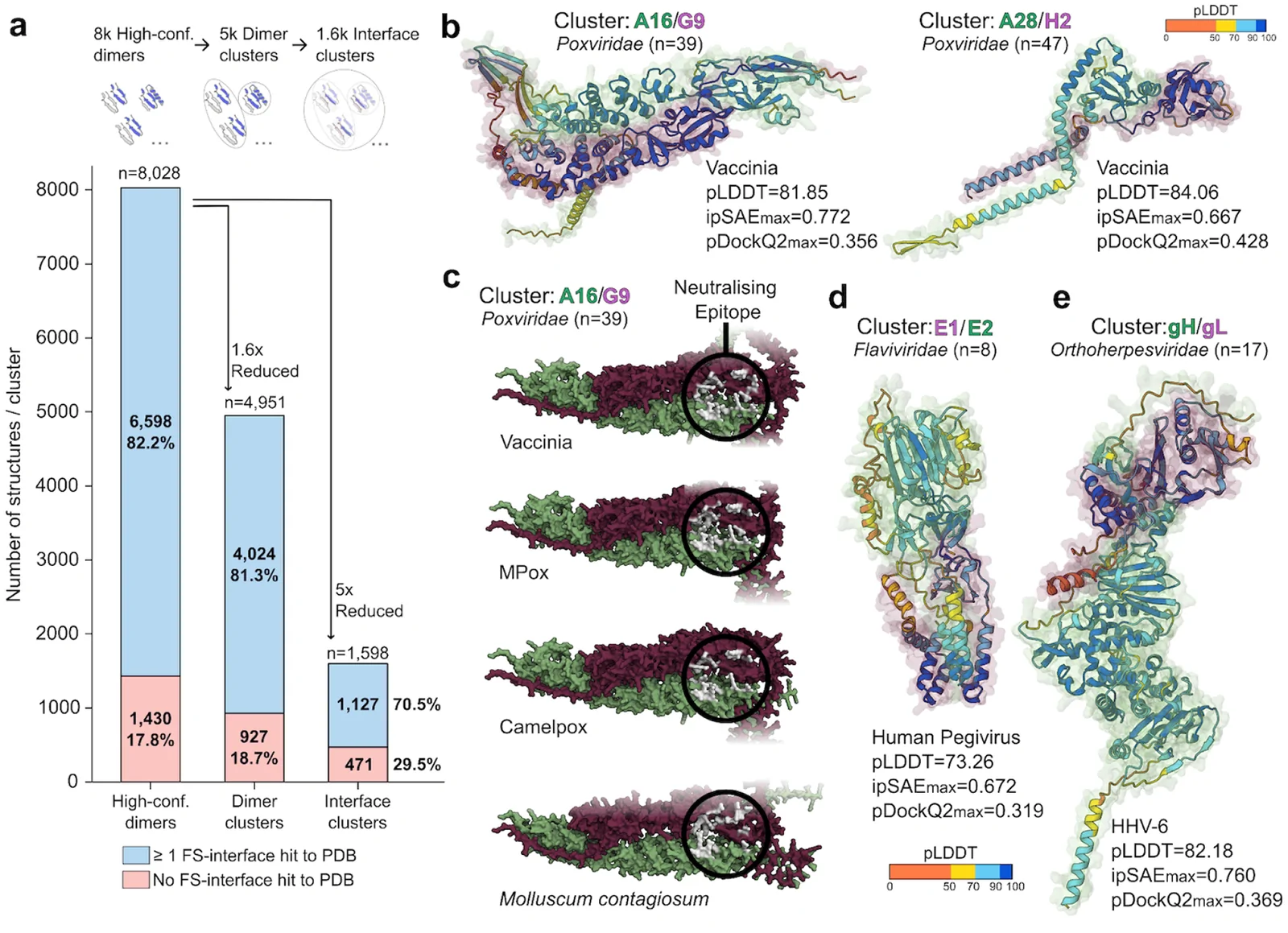
Clustering of the predicted viral interface space and glycoprotein complex example clusters. **a,** Foldseek-Multimer and Foldseek-Interface clustering of 8,028 high-confidence viral dimers (ipSAE_max_ ≥ 0.6; pDockQ2_max_ ≥ 0.23). Foldseek-Multimer clustering reduces the dataset 1.6-fold to 4,951 clusters, while Foldseek-Interface clustering of representatives yields a further 3.1-fold reduction to 1,598 clusters. Coverage of interfaces with a Foldseek-interface hit against PDB experimental interfaces is largely retained after Foldseek-Multimer clustering (82.2% to 81.3%); 471 Foldseek-Interface clusters (29.5%) have no PDB hit. **b,** Vaccinia heterodimers taken from A16/G9 (AF-0000000213241223) and A28/H2 (AF-0000000213229510) interface-clusters. Ribbon diagrams are colour-coded by pLDDT prediction confidence, as denoted in the key, with transparent surface representations highlighting individual chains colour-coded by protein identity, as shown in the complex labels. Annotations provide average pLDDT values and interaction confidence metrics. Cluster labels indicate complex identity, viral taxonomy and, in parentheses, number of members per cluster. **c,** Surface representations of A16/G9 dimers from diverse poxviruses (top to bottom: AF-0000000213241223, AF-0000000213009765, AF-0000000213150491, AF-0000000212886881). A structurally conserved quaternary neutralizing epitope is highlighted in light grey. Surfaces are colour-coded by protein identity as indicated **d,e,** Ribbon diagrams of glycoprotein heteromers from interface-clusters of E1/E2 (*Flaviviridae*; AF-0000000212089925) and gH/gL (*Orthoherpesviridae*). HHV-6: human herpesvirus 6 (AF-0000000212432428).

**Figure 5.**
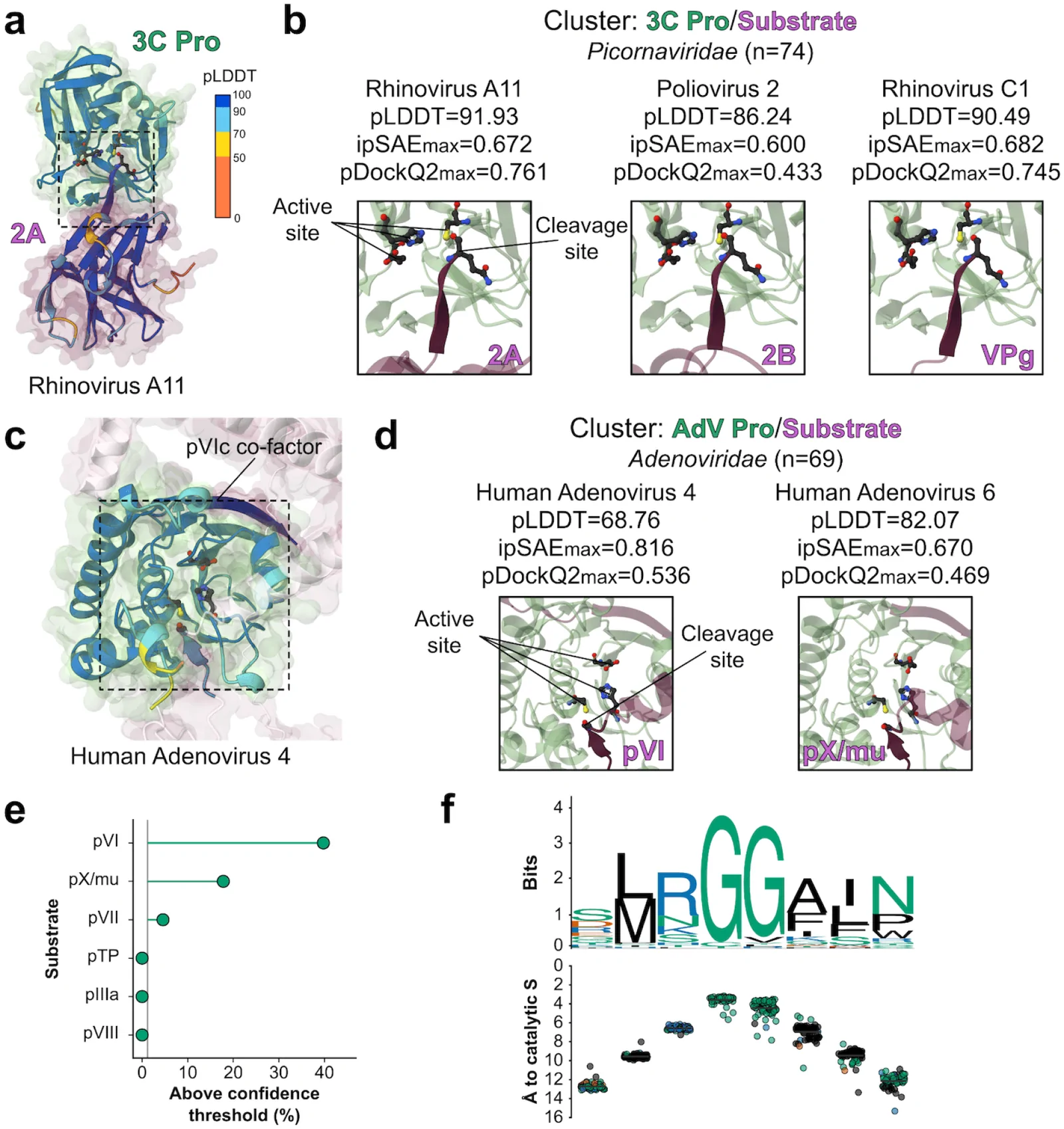
Modelling enzymatic activity of viral proteases. Highlighted residues in chain:number(s) notation. **a,** Ribbon diagram of the rhinovirus A11 3C protease in complex with its substrate 2A (AF-0000000212056371; A:145/B:40,71,147), coloured and annotated as in Fig. 4b; the dashed box indicates the area zoomed in the subsequent panel. **b,** Zoomed views of the 3C protease active site engaged with the substrate cleavage site, for rhinovirus A11 (substrate = 2A; A:141–145/B:40,71,147), poliovirus 2 (substrate = 2B; AF-0000000212064153; A:93–97/B:40,71,147) and rhinovirus C1 (substrate = VPg; AF-0000000212064278; A:18–22/B:40,71,147). In the zoomed views, ribbons show chain colour-coding only; the catalytic triad and the substrate P1 residue are drawn as sticks with heteroatom colouring, and everything beyond the cleavage site is shown in transparency. Cluster labels as in Fig. 4. **c,** Ribbon diagram of the human adenovirus (AdV) 4 protease in complex with its substrate pVI (AF-0000000212158930; A:29–33,230–242/B:56,73,123). The C-terminus of pVI (pVIc) is docked against the protease, recapitulating co-factor interactions. For clarity, low-pLDDT regions of pVI that do not contact the protease are faded to white. **d,** Zoomed views of the AdV protease active site, for human AdV 4 (substrate = pVI; A:29–34/B:56,73,123) and human AdV 6 (substrate = pX/mu; AF-0000000212195305; A:54,71,122/B:47–52), displayed as in b. **e,** Proportion of AdV protease/substrate predictions crossing our confidence thresholds for six classical substrates; the grey line indicates the baseline frequency for non-substrate proteins (1.2%). **f,** Sequence logo (upper) and matched per-residue distance from the catalytic sulfur (lower), for structurally aligned substrates in complex with the AdV protease (n=65 substrate/active site pairs).

## Data Availability

High-confidence dimer, and all monomer predictions are available as individual entry pages through AlphaFold Database at alphafold.ebi.ac.uk. Dimers not passing the high-confidence threshold are provided at ftp.ebi.ac.uk/pub/databases/alphafold/collaborations/nvda/. The folding pipeline is available at github.com/NVIDIA-BioNeMo/BioNeMo-Structure-Prediction-Pipeline.

## References

[R1] Akey David L., Brown W. Clay, Dutta Somnath, 2014. “Flavivirus NS1 Structures Reveal Surfaces for Associations with Membranes and the Immune System.” Science (New York, N.Y.) 343 (6173): 881–885. 10.1126/science.1247749 PMC4263348 24505133

[R2] Baldo A. M., and McClure M. A.. 1999. “Evolution and Horizontal Transfer of dUTPase-Encoding Genes in Viruses and Their Hosts.” Journal of Virology 73 (9): 7710–7721. 10.1128/jvi.73.9.7710-7721.1999 PMC104298 10438861

[R3] Baniecki Mary Lynn, McGrath William J., and Mangel Walter F.. 2013. “Regulation of a Viral Proteinase by a Peptide and DNA in One-Dimensional Space: III. Atomic Resolution Structure of the Nascent Form of the Adenovirus Proteinase.” The Journal of Biological Chemistry 288 (3): 2081–2091. 10.1074/jbc.M112.407429 PMC3548514 23043139

[R4] Bartenschlager Ralf, Lohmann Volker, and Penin Francois. 2013. “The Molecular and Structural Basis of Advanced Antiviral Therapy for Hepatitis C Virus Infection.” Nature Reviews. Microbiology 11 (7): 482–496. 10.1038/nrmicro3046 23748342

[R5] Bertoni Damian, Tsenkov Maxim, Magana Paulyna, 2026. “AlphaFold Protein Structure Database 2025: A Redesigned Interface and Updated Structural Coverage.” Nucleic Acids Research 54 (D1): D358–D362. 10.1093/nar/gkaf1226 PMC12807749 41273079

[R6] Boon Johan A. den, Nishikiori Masaki, Zhan Hong, and Ahlquist Paul. 2024. “Positive-Strand RNA Virus Genome Replication Organelles: Structure, Assembly, Control.” Trends in Genetics : TIG 40 (8): 681–693. 10.1016/j.tig.2024.04.003 PMC13208914 38724328

[R7] Burley Stephen K., Berman Helen M., Bhikadiya Charmi, 2019. “Protein Data Bank: The Single Global Archive for 3D Macromolecular Structure Data.” Nucleic Acids Research 47 (D1): D520–D528. 10.1093/nar/gky949 PMC6324056 30357364

[R8] Chen Rubing, Mukhopadhyay Suchetana, Merits Andres, 2018. “ICTV Virus Taxonomy Profile: Togaviridae.” The Journal of General Virology 99 (6): 761–762. 10.1099/jgv.0.001072 PMC12662122 29745869

[R9] Chikhi Rayan, Lemane Téo, Loll-Krippleber Raphaël, 2025. “Logan: Planetary-Scale Genome Assembly Surveys Life’s Diversity.” In bioRxiv. September 1. 10.1101/2024.07.30.605881.

[R10] Castro De, Edouard Chantal Hulo, Masson Patrick, Auchincloss Andrea, Bridge Alan, and Le Mercier Philippe. 2024. “ViralZone 2024 Provides Higher-Resolution Images and Advanced Virus-Specific Resources.” Nucleic Acids Research 52 (D1): D817–D821. 10.1093/nar/gkad946 PMC10767872 37897348

[R11] Didi Kieran, Sohani Prashant, Berressem Fabian, 2026. “Efficient Protein Structure Prediction from Compact Computers to Datacenters with OpenFold-TRT.” In bioRxiv. March 15. 10.64898/2026.03.11.711233.

[R12] Ding J., McGrath W. J., Sweet R. M., and Mangel W. F.. 1996. “Crystal Structure of the Human Adenovirus Proteinase with Its 11 Amino Acid Cofactor.” The EMBO Journal 15 (8): 1778–1783. PMC450093 8617222

[R13] Dunbrack Roland L. Jr. 2025. “Rēs ipSAE Loquuntur: What’s Wrong with AlphaFold’s ipTM Score and How to Fix It.” bioRxiv: The Preprint Server for Biology, February 14, 2025.02.10.637595.

[R14] Evans Richard, Michael O’Neill Alexander Pritzel, 2021. “Protein Complex Prediction with AlphaFold-Multimer.” In bioRxiv. BioRxiv. 10.1101/2021.10.04.463034.

[R15] Faysal K. M. Rifat, Walsh James C., Renner Nadine, 2024. “Pharmacologic Hyperstabilisation of the HIV-1 Capsid Lattice Induces Capsid Failure.” eLife 13 (February): e83605. 10.7554/eLife.83605 PMC10863983 38347802

[R16] Firth Andrew E. 2014. “Mapping Overlapping Functional Elements Embedded within the Protein-Coding Regions of RNA Viruses.” Nucleic Acids Research 42 (20): 12425–12439. 10.1093/nar/gku981 PMC4227794 25326325

[R17] Ge Yingwei, Zhou Lijie, Fu Yesheng, 2024. “Caspase-2 Is a Condensate-Mediated Deubiquitinase in Protein Quality Control.” Nature Cell Biology 26 (11): 1943–1957. 10.1038/s41556-024-01522-8 PMC11567894 39482354

[R18] Guirimand Thibaut, Delmotte Stéphane, and Navratil Vincent. 2015. “VirHostNet 2.0: Surfing on the Web of Virus/host Molecular Interactions Data.” Nucleic Acids Research 43 (Database issue): D583–7. 10.1093/nar/gku1121 PMC4383936 25392406

[R19] Haas Meryl, Mills Jake, Kelley Cynthia, 2025. “Integrative Structure of Norovirus NS3 Suggests a Role in RNA Transport.” In bioRxiv. BioRxiv. 10.1101/2025.06.17.659504. PMC13683342 42901927

[R20] Han Yewon, Tsenkov Maxim I., Venanzi Niccolò A. E., 2026. “AlphaFold Database Expands to Proteome-Scale Quaternary Structures.” In bioRxiv. BioRxiv, March 29. 10.64898/2026.03.27.714458.

[R21] Hermjakob Henning, Montecchi-Palazzi Luisa, Lewington Chris, 2004. “IntAct: An Open Source Molecular Interaction Database.” Nucleic Acids Research 32 (Database issue): D452–5. 10.1093/nar/gkh052 PMC308786 14681455

[R22] Humphreys Ian R., Pei Jimin, Baek Minkyung, 2021. “Computed Structures of Core Eukaryotic Protein Complexes.” Science (New York, N.Y.) 374 (6573): eabm4805. 10.1126/science.abm4805 PMC7612107 34762488

[R23] Jayaraman Bhargavi, Smith Amber M., Fernandes Jason D., and Frankel Alan D.. 2016. “Oligomeric Viral Proteins: Small in Size, Large in Presence.” Critical Reviews in Biochemistry and Molecular Biology 51 (5): 379–394. 10.1080/10409238.2016.1215406 PMC5088105 27685368

[R24] Jumper John, Evans Richard, Pritzel Alexander, 2021. “Highly Accurate Protein Structure Prediction with AlphaFold.” Nature 596 (7873): 583–589. 10.1038/s41586-021-03819-2 PMC8371605 34265844

[R25] J Woolhouse Mark E., Adair Kyle, and Brierley Liam. 2013. “RNA Viruses: A Case Study of the Biology of Emerging Infectious Diseases.” Microbiology Spectrum 1 (1): 1–11. 10.1128/microbiolspec.OH-0001-2012 PMC6157708 26184815

[R26] Kallenborn Felix, Chacon Alejandro, Hundt Christian, 2025. “GPU-Accelerated Homology Search with MMseqs2.” Nature Methods 22 (10): 2024–2027. 10.1038/s41592-025-02819-8 PMC12510879 40968302

[R27] Kaur Gurpreet, Crawford Sue E., Cregeen Sara Javornik, 2026. “Overcoming Host Restrictions to Enable Continuous Passaging of GII.3 Human Norovirus in Human Intestinal Enteroids.” Science Advances 12 (6): eaeb0455. 10.1126/sciadv.aeb0455 PMC12871457 41637508

[R28] Kim Rachel Seongeun, Karin Eli Levy, Mirdita Milot, Chikhi Rayan, and Steinegger Martin. 2025. “BFVD-a Large Repository of Predicted Viral Protein Structures.” Nucleic Acids Research 53 (D1): D340–D347. 10.1093/nar/gkae1119 PMC11701548 39574394

[R29] Kim Rachel Seongeun, Pimenova Olga, Eli Levy Karin Milot Mirdita, and Steinegger Martin. 2026. “BFVD v3–UniProt-Complete, Improved Viral Protein Structure Predictions.” In bioRxiv. BioRxiv, September 18. 10.64898/2026.09.16.752260.

[R30] Kim Woosub, Mirdita Milot, Karin Eli Levy, 2025. “Rapid and Sensitive Protein Complex Alignment with Foldseek-Multimer.” Nature Methods 22 (3): 469–472. 10.1038/s41592-025-02593-7 PMC11903335 39910251

[R31] Lasso Gorka, Mayer Sandra V., Winkelmann Evandro R., 2019. “A Structure-Informed Atlas of Human-Virus Interactions.” Cell 178 (6): 1526–1541.e16. 10.1016/j.cell.2019.08.005 PMC6736651 31474372

[R32] Li Jiachen, Ma Sen, Yang Runze, Xu Jinrui, Wang Yaxin, and Ye Sheng. 2024. “Structural Definition of Pseudorabies Virus dUTPase Reveals a Novel Folding Dimer in the Herpesvirus Family.” International Journal of Biological Macromolecules 280 (Pt 1): 135696. 10.1016/j.ijbiomac.2024.135696 39284464

[R33] Lin Chang Sheng-Huei, Li Ching-An, Wang Chun-Hsiung, 2026. “Cryo-EM Structure of the Vaccinia Virus Entry Fusion Complex Reveals a Multicomponent Fusion Machinery.” Science Advances 12 (3): eaec0254. 10.1126/sciadv.aec0254 PMC12802840 41533782

[R34] Link John O., Rhee Martin S., Tse Winston C., 2020. “Clinical Targeting of HIV Capsid Protein with a Long-Acting Small Molecule.” Nature 584 (7822): 614–618. 10.1038/s41586-020-2443-1 PMC8188729 32612233

[R35] Lithgo Ryan M., Tomlinson Charles W. E., Fairhead Michael, 2024. “Crystallographic Fragment Screen of Coxsackievirus A16 2A Protease Identifies New Opportunities for the Development of Broad-Spectrum Anti-Enterovirals.” In bioRxiv. April 29. 10.1101/2024.04.29.591684.

[R36] Litvin Ulad, Lytras Spyros, Jack Alexander, Robertson David L., Hughes Joseph, and Grove Joe. 2025. “Viro3D: A Comprehensive Database of Virus Protein Structure Predictions.” Molecular Systems Biology 21 (11): 1599–1617. 10.1038/s44320-025-00147-9 PMC12583693 40958060

[R37] Li Xiao-Dong, Sun Lijun, Seth Rashu B., Pineda Gabriel, and Chen Zhijian J.. 2005. “Hepatitis C Virus Protease NS3/4A Cleaves Mitochondrial Antiviral Signaling Protein off the Mitochondria to Evade Innate Immunity.” Proceedings of the National Academy of Sciences of the United States of America 102 (49): 17717–17722. 10.1073/pnas.0508531102 PMC1308909 16301520

[R38] Lytras Spyros, Ghafari Mahan, and Grove Joe. 2026. “Studying the Deep Evolution of Viruses in the Era of Artificial Intelligence Structure Prediction.” Annual Review of Virology, ahead of print, June 18. 10.1146/annurev-virology-100424-122154. PMC7619455 42314068

[R39] McCool Ryan S., Acreman Cory M., Powell Abigail E., 2026. “Structure and Immunogenicity of an Engineered Soluble Prefusion-Stabilized EBV gB Antigen.” Nature Communications 17 (1): 1197. 10.1038/s41467-025-67969-x PMC12859058 41484092

[R40] McGrath William J., Ding Jianzhong, Didwania Aashish, Sweet Robert M., and Mangel Walter F.. 2003. “Crystallographic Structure at 1.6-Å Resolution of the Human Adenovirus Proteinase in a Covalent Complex with Its 11-Amino-Acid Peptide Cofactor: Insights on a New Fold.” Biochimica et Biophysica Acta 1648 (1–2): 1–11. 10.1016/s1570-9639(03)00024-4 12758141

[R41] McLellan Jason S., Chen Man, Joyce M. Gordon, 2013. “Structure-Based Design of a Fusion Glycoprotein Vaccine for Respiratory Syncytial Virus.” Science (New York, N.Y.) 342 (6158): 592–598. 10.1126/science.1243283 PMC4461862 24179220

[R42] Meola Annalisa, Vernuccio Riccardo, Battini Leandro, 2025. “Structural Basis of Poxvirus Fusion Regulation and Anti-A16/G9 Antibody-Mediated Neutralization and Protection.” Cell 188 (22): 6266–6282.e18. 10.1016/j.cell.2025.07.040 40865523

[R43] Mifsud Jonathon C. O., Lytras Spyros, Oliver Michael R., 2024. “Mapping Glycoprotein Structure Reveals Flaviviridae Evolutionary History.” Nature 633 (8030): 695–703. 10.1038/s41586-024-07899-8 PMC11410658 39232167

[R44] Muratore Giulia, Goracci Laura, Mercorelli Beatrice, 2012. “Small Molecule Inhibitors of Influenza A and B Viruses That Act by Disrupting Subunit Interactions of the Viral Polymerase.” Proceedings of the National Academy of Sciences of the United States of America 109 (16): 6247–6252. 10.1073/pnas.1119817109 PMC3341009 22474359

[R45] Nelen Jochem, Khan Omeir, Adams Etowah, 2026. “The First OpenBind Release: An Open Experimental Structure–affinity Dataset and Benchmark for Structure-Based AI.” In bioRxiv. BioRxiv, August 28. 10.64898/2026.08.27.747600.

[R46] Nomburg Jason, Doherty Erin E., Price Nathan, Bellieny-Rabelo Daniel, Zhu Yong K., and Doudna Jennifer A.. 2024. “Birth of Protein Folds and Functions in the Virome.” Nature 633 (8030): 710–717. 10.1038/s41586-024-07809-y PMC11410667 39187718

[R47] Odai Roni, Leemann Michèle, Al-Murad Tamim, 2025. “The Viral AlphaFold Database of Monomers and Homodimers Reveals Conserved Protein Folds in Viruses of Bacteria, Archaea, and Eukaryotes.” Science Advances 11 (40): eadz8560. 10.1126/sciadv.adz8560 PMC12487901 41032608

[R48] Paciello Ida, Rundlet Emily J., Zhou Ling, 2025. “Antigen-Agnostic Identification of Poxvirus Broadly Neutralizing Antibodies Targeting OPG153.” Science Translational Medicine 17 (828): eaeb3840. 10.1126/scitranslmed.aeb3840 41370398

[R49] Prasad B. V. Venkataram, and Schmid Michael F.. 2012. “Principles of Virus Structural Organization.” Advances in Experimental Medicine and Biology 726: 17–47. 10.1007/978-1-4614-0980-9_3 PMC3767311 22297509

[R50] Qi Xianzhi, Ye Cheng, Liang Jianqiang, 2026. “Atlas of Predicted Protein Complex Structures across Kingdoms.” Nature Communications 17 (1). 10.1038/s41467-026-70884-4. PMC13181095 41882029

[R51] Rawlings Neil D., and Bateman Alex. 2019. “Origins of Peptidases.” Biochimie 166 (November): 4–18. 10.1016/j.biochi.2019.07.026 PMC7117030 31377195

[R52] Labra Oscar Rojas, Montoya-Munoz David S., Santoyo-Rivera Nelly, 2026. “FoldaVirus, a Knowledge-Based Icosahedral Capsid Prediction Tool Using AlphaFold.” Proceedings of the National Academy of Sciences of the United States of America 123 (38): e2613741123. 10.1073/pnas.2613741123 PMC13598199 42752236

[R53] Sanders Rogier W., and Moore John P.. 2021. “Virus Vaccines: Proteins Prefer Prolines.” Cell Host & Microbe 29 (3): 327–333. 10.1016/j.chom.2021.02.002 PMC7945883 33705704

[R54] Schmid Ernst W., Zhu Helen, Ryu Eunjin, 2025. “Proteome-Wide in Silico Screening for Human Protein-Protein Interactions.” In bioRxiv. November 12. 10.1101/2025.11.10.687652.

[R55] Sehnal David, Bittrich Sebastian, Deshpande Mandar, 2021. “Mol* Viewer: Modern Web App for 3D Visualization and Analysis of Large Biomolecular Structures.” Nucleic Acids Research 49 (W1): W431–W437. 10.1093/nar/gkab314 PMC8262734 33956157

[R56] Seipelt J., Guarné A., Bergmann E., 1999. “The Structures of Picornaviral Proteinases.” Virus Research 62 (2): 159–168. 10.1016/s0168-1702(99)00043-x 10507325

[R57] Shah Priya S., Beesabathuni Nitin S., Fishburn Adam T., 2022. “Systems Biology of Virus-Host Protein Interactions: From Hypothesis Generation to Mechanisms of Replication and Pathogenesis.” Annual Review of Virology 9 (1): 397–415. 10.1146/annurev-virology-100520-011851 PMC10150767 35576593

[R58] Simmonds Peter, Butković Anamarija, Grove Joe, 2025. “Taxonomic Expansion and Reorganization of Flaviviridae.” Nature Microbiology 10 (11): 3026–3037. 10.1038/s41564-025-02134-0 41174177

[R59] Soh Timothy K., Ognibene Sofia, Sanders Saskia, Robin Schäper Benedikt B. Kaufer, and Bosse Jens B.. 2024. “A Proteome-Wide Structural Systems Approach Reveals Insights into Protein Families of All Human Herpesviruses.” Nature Communications 15 (1): 10230. 10.1038/s41467-024-54668-2 PMC11599850 39592652

[R60] Stark Chris, Breitkreutz Bobby-Joe, Reguly Teresa, Boucher Lorrie, Breitkreutz Ashton, and Tyers Mike. 2006. “BioGRID: A General Repository for Interaction Datasets.” Nucleic Acids Research 34 (Database issue): D535–9. 10.1093/nar/gkj109 PMC1347471 16381927

[R61] Stray Stephen J., Bourne Christina R., Punna Sreenivas, Lewis Warren G., Finn M. G., and Zlotnick Adam. 2005. “A Heteroaryldihydropyrimidine Activates and Can Misdirect Hepatitis B Virus Capsid Assembly.” Proceedings of the National Academy of Sciences of the United States of America 102 (23): 8138–8143. 10.1073/pnas.0409732102 PMC1149411 15928089

[R62] Strom Joelle Morgan, Cha Sooyoung, Kim Rachel Seongeun, 2026. “Foldseek-Interface Reveals a Protein Interface Universe far from Complete.” In bioRxiv. BioRxiv, August 25. 10.64898/2026.08.24.746585.

[R63] Szklarczyk Damian, Nastou Katerina, Koutrouli Mikaela, 2025. “The STRING Database in 2025: Protein Networks with Directionality of Regulation.” Nucleic Acids Research 53 (D1): D730–D737. 10.1093/nar/gkae1113 PMC11701646 39558183

[R64] Tang Huamin, Serada Satoshi, Kawabata Akiko, 2013. “CD134 Is a Cellular Receptor Specific for Human Herpesvirus-6B Entry.” Proceedings of the National Academy of Sciences of the United States of America 110 (22): 9096–9099. 10.1073/pnas.1305187110 PMC3670305 23674671

[R65] Tareen Ammar, and Kinney Justin B.. 2020. “Logomaker: Beautiful Sequence Logos in Python.” Bioinformatics (Oxford, England) 36 (7): 2272–2274. 10.1093/bioinformatics/btz921 PMC7141850 31821414

[R66] Todor Horia, Kim Lili M., Jänes Jürgen, 2026. “Predicting the Protein Interaction Landscape of a Free-Living Bacterium with Pooled-AlphaFold3.” Molecular Systems Biology 22 (4): 497–518. 10.1038/s44320-026-00189-7 PMC13047044 41559189

[R67] UniProt Consortium. 2025. “UniProt: The Universal Protein Knowledgebase in 2025.” Nucleic Acids Research 53 (D1): D609–D617. 10.1093/nar/gkae1010 PMC11701636 39552041

[R68] Urban Martin, Cuzick Alayne, Seager James, 2025. “PHI-Base - the Multi-Species Pathogen-Host Interaction Database in 2025.” Nucleic Acids Research 53 (D1): D826–D838. 10.1093/nar/gkae1084 PMC11701570 39588765

[R69] Veizaj Dejvid, Poole David A. 3rd, Ka Lei Cheung, 2026. “Molecular Modeling of Protein-Peptide Complexes Mimicking Factor Xa-Prothrombin Using AlphaFold-Multimer and Molecular Dynamics Simulations for Functional Prediction.” Journal of Chemical Information and Modeling 66 (11): 6565–6575. 10.1021/acs.jcim.5c02774 42176283

[R70] Woolhouse Mark, Scott Fiona, Hudson Zoe, Howey Richard, and Chase-Topping Margo. 2012. “Human Viruses: Discovery and Emergence.” Philosophical Transactions of the Royal Society of London. Series B, Biological Sciences 367 (1604): 2864–2871. 10.1098/rstb.2011.0354 PMC3427559 22966141

[R71] Wrapp Daniel, Wang Nianshuang, Corbett Kizzmekia S., 2020. “Cryo-EM Structure of the 2019-nCoV Spike in the Prefusion Conformation.” Science (New York, N.Y.) 367 (6483): 1260–1263. 10.1126/science.abb2507 PMC7164637 32075877

[R72] Yang Fanli, Lin Sheng, Chen Zimin, 2023. “Structural Basis of Poxvirus A16/G9 Binding for Sub-Complex Formation.” Emerging Microbes & Infections 12 (1): 2179351. 10.1080/22221751.2023.2179351 PMC9980159 36757688

[R73] Yu Huibin, Resch Wolfgang, and Moss Bernard. 2025. “Poxvirus Structural Biology for Application to Vaccine Design.” Trends in Immunology 46 (6): 455–470. 10.1016/j.it.2025.04.002 PMC12167143 40340168

[R74] Zhang Jing, Humphreys Ian R., Pei Jimin, 2025. “Predicting Protein-Protein Interactions in the Human Proteome.” Science (New York, N.Y.) 390 (6771): eadt1630. 10.1126/science.adt1630 PMC13281779 40997207

[R75] Zhang Tianyu, Kraft Julian, Soh Timothy K., Kansy Malte, Jonsson Ingvar, and Bosse Jens B.. 2026. “Systematic Validation of AlphaFold-Predicted Interactomes with LUCIA.” In bioRxiv. BioRxiv, April 4. 10.64898/2026.04.02.716051.

[R76] Zhu Na, Zhang Dingyu, Wang Wenling, 2020. “A Novel Coronavirus from Patients with Pneumonia in China, 2019.” The New England Journal of Medicine 382 (8): 727–733. 10.1056/NEJMoa2001017 PMC7092803 31978945

[R77] Zhu Wensi, Shenoy Aditi, Kundrotas Petras, and Elofsson Arne. 2023. “Evaluation of AlphaFold-Multimer Prediction on Multi-Chain Protein Complexes.” Bioinformatics (Oxford, England) 39 (7): btad424. 10.1093/bioinformatics/btad424 PMC10348836 37405868

[R78] Zlobin Alexander, and Golovin Andrey. 2022. “Between Protein Fold and Nucleophile Identity: Multiscale Modeling of the TEV Protease Enzyme-Substrate Complex.” ACS Omega 7 (44): 40279–40292. 10.1021/acsomega.2c05201 PMC9647873 36385818

[R79] Zlotnick Adam, and Mukhopadhyay Suchetana. 2011. “Virus Assembly, Allostery and Antivirals.” Trends in Microbiology 19 (1): 14–23. 10.1016/j.tim.2010.11.003 PMC3026312 21163649

