# Supplementary material for: Systematic exploration of predicted quaternary structures within pandemic-relevant viral proteomes
Source: bioRxiv. 2026 Oct 1:2026.09.29.755321. Preprint. [Version 1] doi: 10.64898/2026.09.29.755321 (PMC13648588; doi:10.64898/2026.09.29.755321)
Supplement: Supplementary file 1 [file NIHPP2026.09.29.755321V1-supplement-1.pdf]

bioRxiv preprint doi: https://doi.org/10.64898/2026.09.29.755321; this version posted October 1, 2026. The copyright holder for this preprint (which was not certified by peer review) is the author/funder, who has granted bioRxiv a license to display the preprint in perpetuity. It is made available under a CC-BY 4.0 International license.

Supplementary Figures

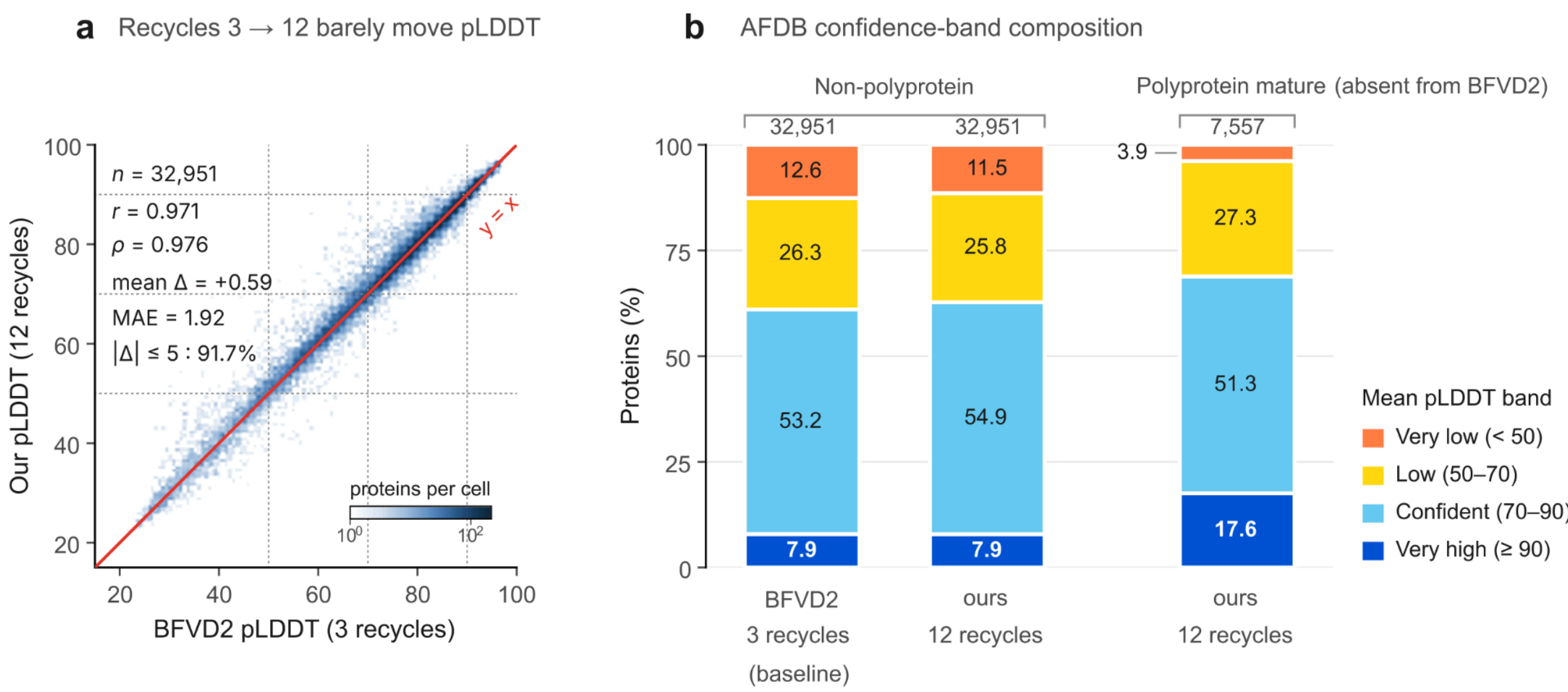

Supplementary Figure 1. Monomer confidence is robust to recycling depth and elevated for mature polyprotein products. a, Of the 41,869 post-processed monomeric predicted structures,32,957 were mappable to BFVD v3 ((R. S. Kim et al. 2025; Litvin et al. 2025; Odai et al. 2025; R. S. Kim et al. 2026)). Mean pLDDT for 32,951 non-polyprotein viral proteins predicted with three recycles (BFVD v3) or 12 recycles (used in this study), with otherwise identical ColabFold settings. Predictions were highly concordant (Pearson r = 0.9708; Spearman ρ = 0.9756; mean absolute difference, 1.92 pLDDT), with a mean increase of 0.59 pLDDT at 12 recycles; 91.72% differed by ≤5 pLDDT and 91.67% remained in the same AlphaFold DB confidence category. Density is shown on a logarithmic scale; the red line denotes $y = x$ and dashed lines mark pLDDT thresholds of 50, 70 and 90. **b,** Distribution across AlphaFold Database confidence categories. Increasing from three to 12 recycles reduced the very-low-confidence fraction by 1.2 percentage points but left the very-high-confidence fraction unchanged (7.89% versus 7.88%). Mature polyprotein products (n = 7,557) were more often very high confidence (17.6% versus 7.9%) and less often very low confidence (3.9% versus 11.5%) than non-polyprotein proteins. BFVD v3 contains no equivalent mature-product set. Numbers denote protein counts and percentages. pLDDT measures model confidence, not structural accuracy.

bioRxiv preprint doi: https://doi.org/10.64898/2026.09.29.755321; this version posted October 1, 2026. The copyright holder for this preprint (which was not certified by peer review) is the author/funder, who has granted bioRxiv a license to display the preprint in perpetuity. It is made available under a CC-BY 4.0 International license.

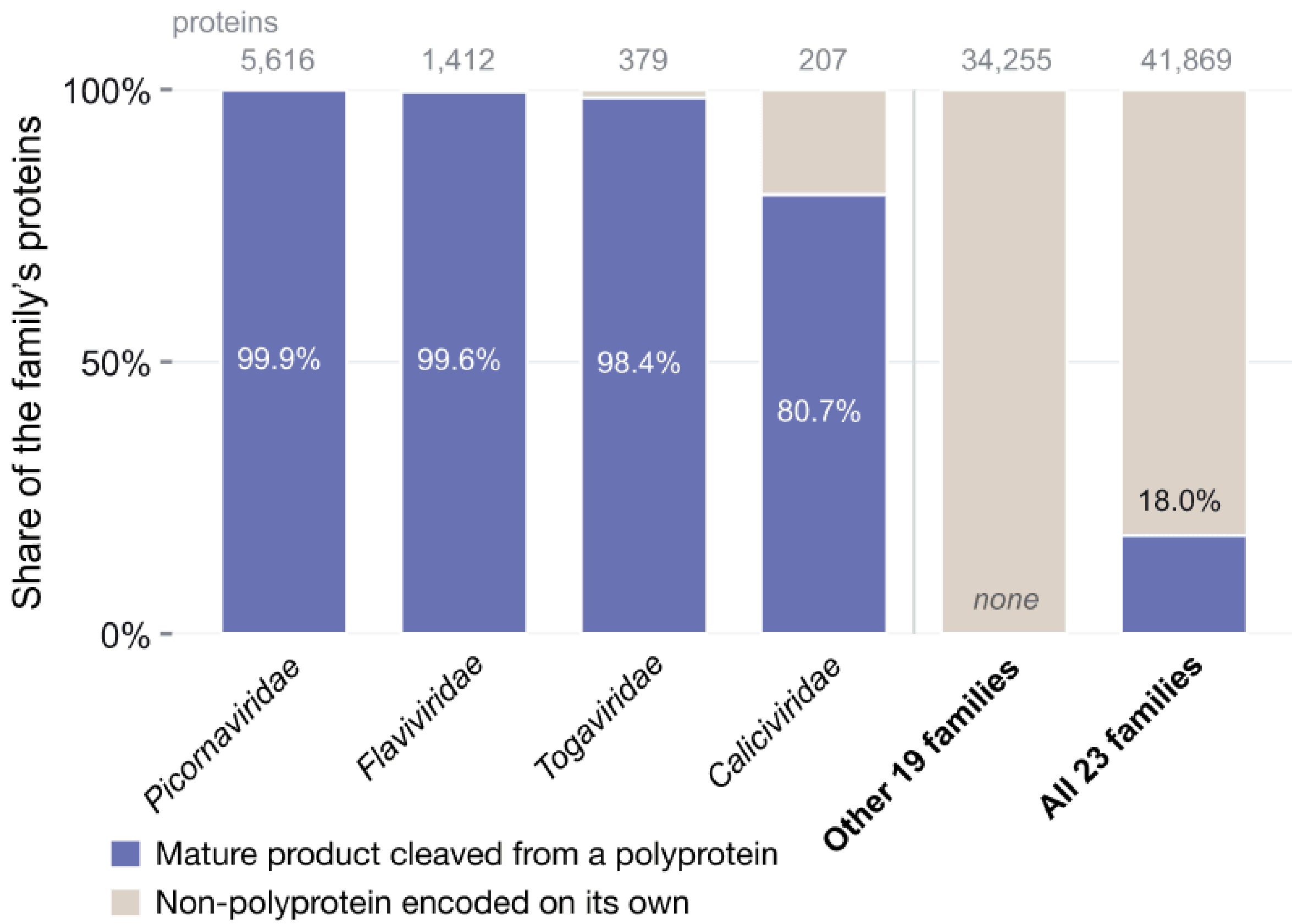

Supplementary Figure 2. Polyprotein content of each viral family. Four of 23 viral families encode polyproteins, which accounts for 81-100% of their proteins. In contrast, the other 19 families, holding 34,160 proteins, do not encode polyproteins. Of the 41,869 post-processed monomeric predicted structures, 7,557 (18.1%) corresponded to polyprotein mature products. Families follow VMR 40, which places *Hepaciviridae* and *Pestiviridae* inside *Flaviviridae*; this merge reduces the number of families with polyproteins from 6 to 4.

bioRxiv preprint doi: https://doi.org/10.64898/2026.09.29.755321; this version posted October 1, 2026. The copyright holder for this preprint (which was not certified by peer review) is the author/funder, who has granted bioRxiv a license to display the preprint in perpetuity. It is made available under a CC-BY 4.0 International license.

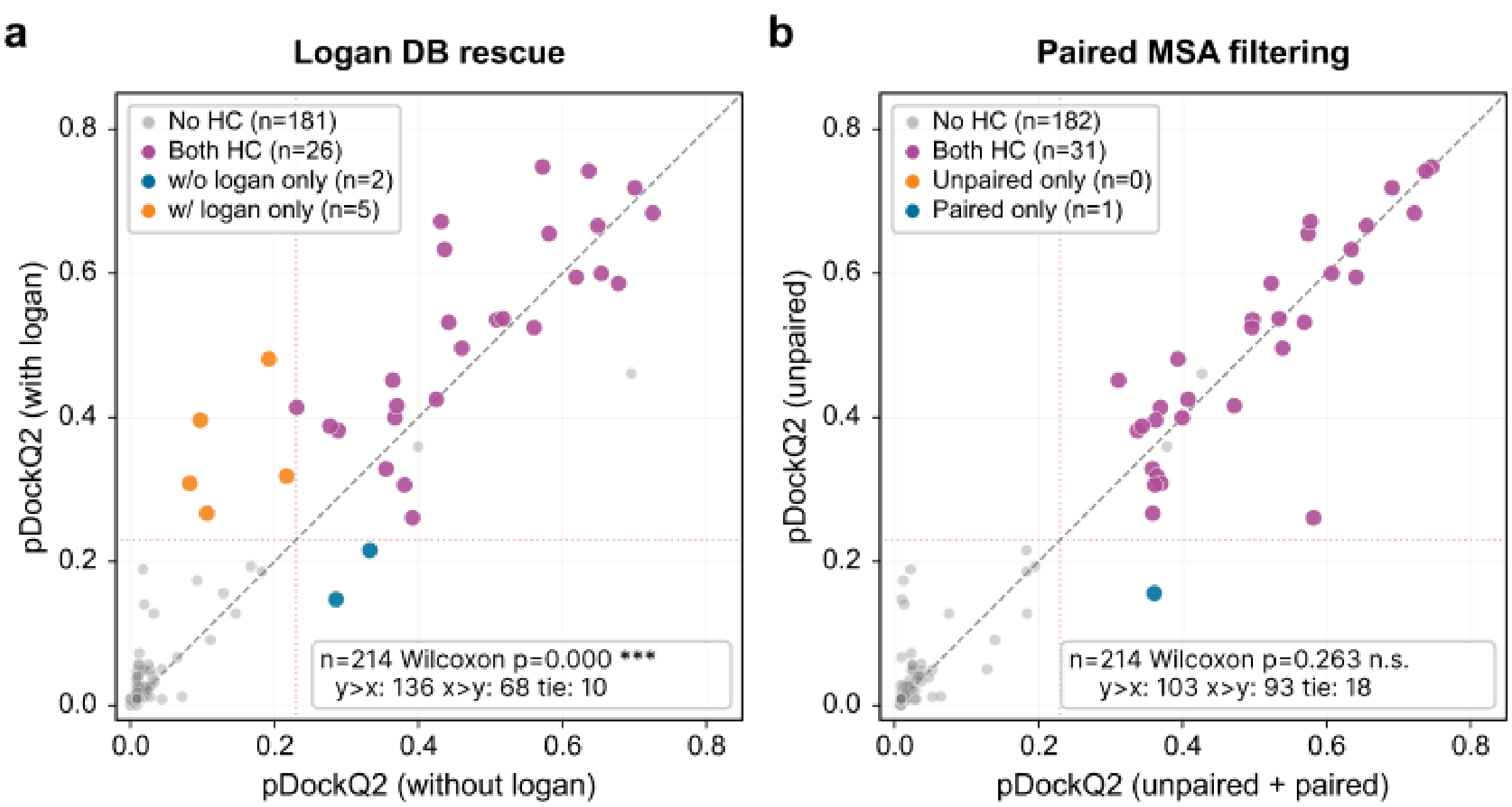

Supplementary Figure 3. pDockQ2-based evaluation of MSA curation strategies. Same analysis as Fig. 2a,b, displaying pDockQ2 rather than ipSAE. High-confidence classification remains defined jointly by ipSAE ≥ 0.6 and pDockQ2 ≥ 0.23; dotted lines mark the pDockQ2 threshold. **a,** Effect of LoganDB enrichment. pDockQ2 was significantly higher with LoganDB enrichment (Wilcoxon signed-rank test, $P < 0.001$), increasing in 136 of 214 benchmark pairs. High-confidence classifications were the same to **Fig. 2a** (both, $n = 26$; 'with Logan' only, $n = 5$; 'without LoganDB' only, $n = 2$). **b,** Effect of paired MSA filtering. pDockQ2 scores were similar between filtered paired-plus-unpaired and unpaired-only MSAs (Wilcoxon signed-rank test, $P = 0.299$), consistent with the ipSAE analysis in **Fig. 2b**.

bioRxiv preprint doi: https://doi.org/10.64898/2026.09.29.755321; this version posted October 1, 2026. The copyright holder for this preprint (which was not certified by peer review) is the author/funder, who has granted bioRxiv a license to display the preprint in perpetuity. It is made available under a CC-BY 4.0 International license.

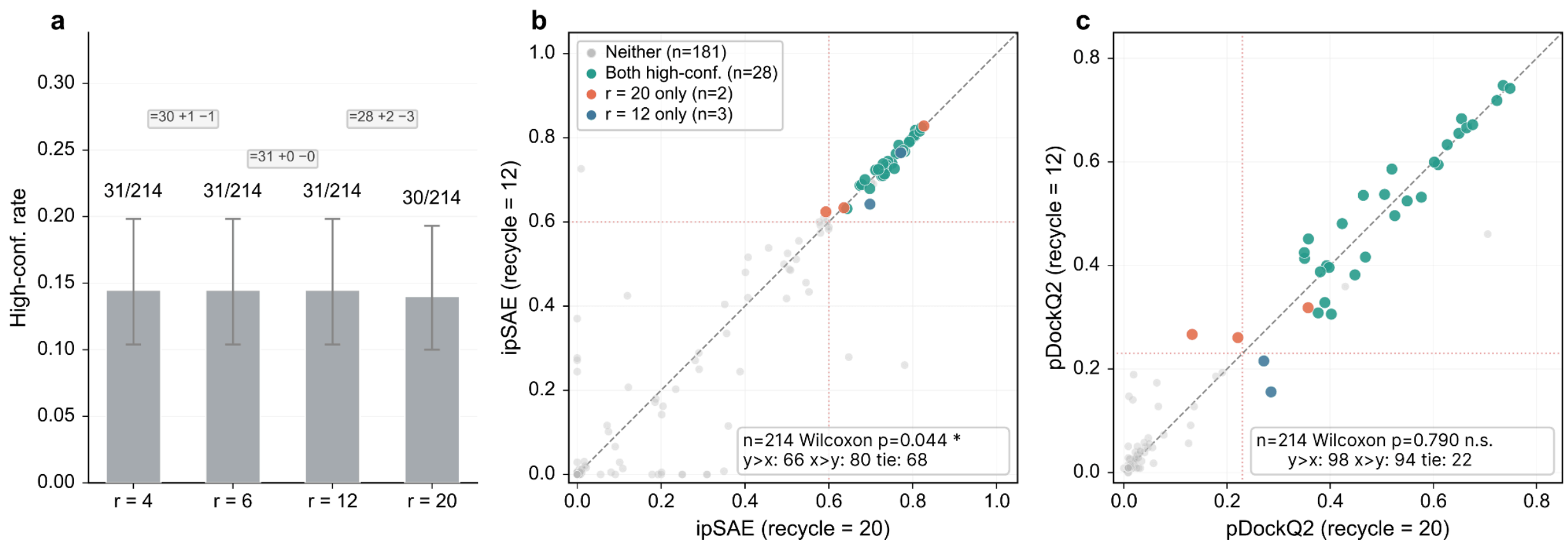

Supplementary Figure 4. High-confidence classification is robust to the number of recycling iterations. **a,** Fraction of high-confidence predictions across 4, 6, 12 and 20 recycling iterations for the unpaired-MSA pipeline (n = 214 evaluable non-phage benchmark pairs). High confidence was defined jointly as ipSAE ≥ 0.6 and pDockQ2 ≥ 0.23. Error bars indicate 95% Wilson confidence intervals. Annotations show the numbers of concordant (=), gained (+) and lost (−) high-confidence pairs between consecutive settings. Counts were nearly invariant (31, 31, 31 and 30), with complete concordance between 6 and 12 recycles. **b,c,** Per-pair comparisons of ipSAE (b) and pDockQ2 (c) at 20 recycles (x axis; highest recycling depth tested) and 12 recycles (y axis; setting used in this study). Points are coloured by joint classification: teal, high confidence at both settings; coral, 20 recycles only; steel blue, 12 recycles only; grey, neither.

bioRxiv preprint doi: https://doi.org/10.64898/2026.09.29.755321; this version posted October 1, 2026. The copyright holder for this preprint (which was not certified by peer review) is the author/funder, who has granted bioRxiv a license to display the preprint in perpetuity. It is made available under a CC-BY 4.0 International license.

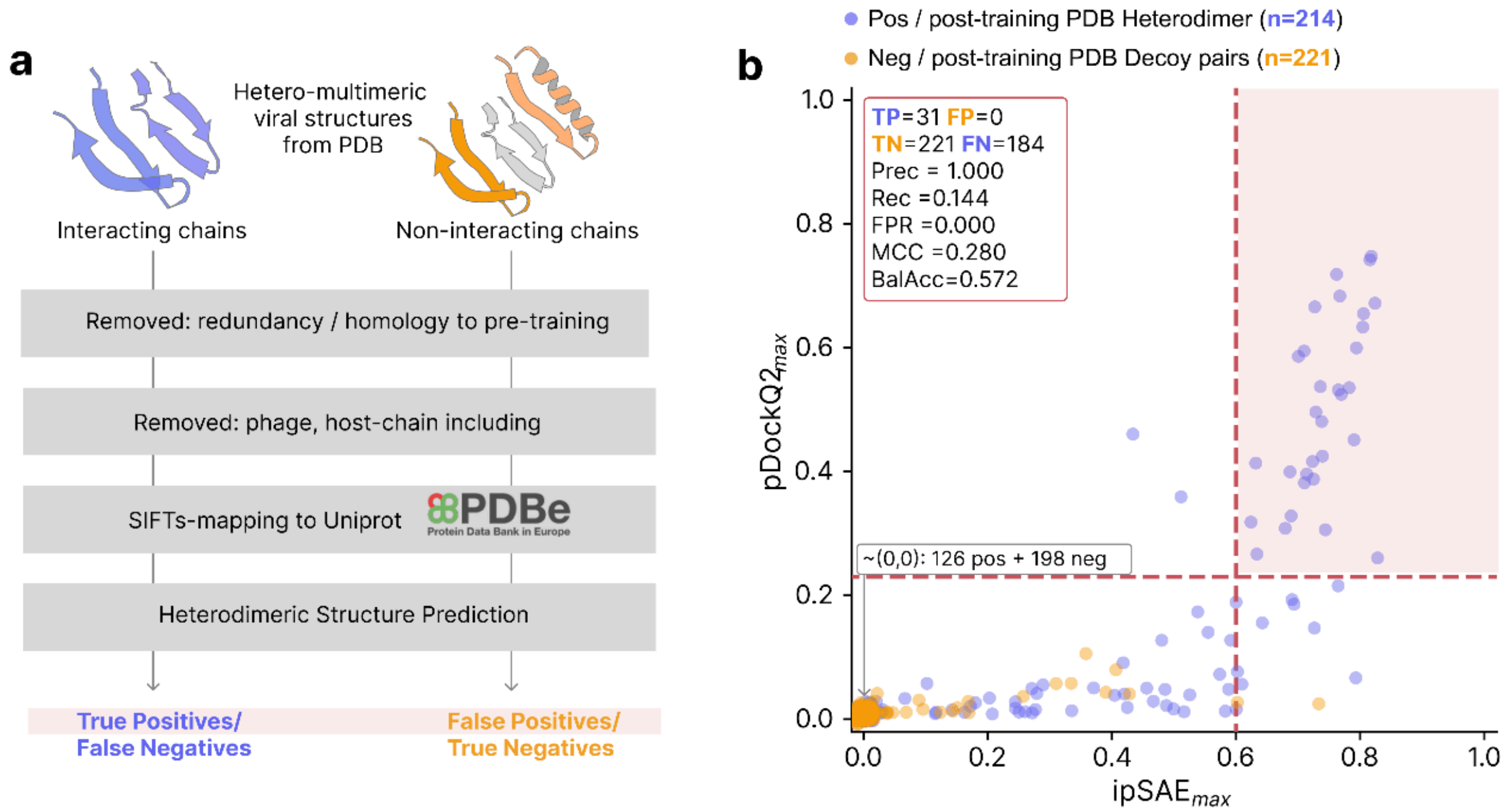

Supplementary Figure 5. Post-training benchmark of the viral dimer prediction pipeline. a, Benchmark workflow. Positive (n = 215) and negative (n = 221) pairs were derived from post-training PDB structures, mapped to UniProt using SIFTS and modelled with our prediction pipeline. Structures similar to pre-training complexes were excluded using Foldseek-Multimer searches (Methods). Classification used 215 positive and 221 evaluable negative pairs. **b,** Joint distribution of $ipSAE_{max}$ and $pDockQ2_{max}$ for positive (purple) and negative (orange) pairs. Each metric is the maximum across the two chain directions. Dashed lines mark the joint high-confidence criterion ($ipSAE_{max} \geq 0.60$ and $pDockQ2_{max} \geq 0.23$). High-confidence positives and negatives are true positives (TP = 31) and false positives (FP = 0), respectively; the remaining evaluable pairs are false negatives (FN = 184) and true negatives (TN = 221). Points near the origin are jittered for visibility; classification uses unmodified scores.

bioRxiv preprint doi: https://doi.org/10.64898/2026.09.29.755321; this version posted October 1, 2026. The copyright holder for this preprint (which was not certified by peer review) is the author/funder, who has granted bioRxiv a license to display the preprint in perpetuity. It is made available under a CC-BY 4.0 International license.

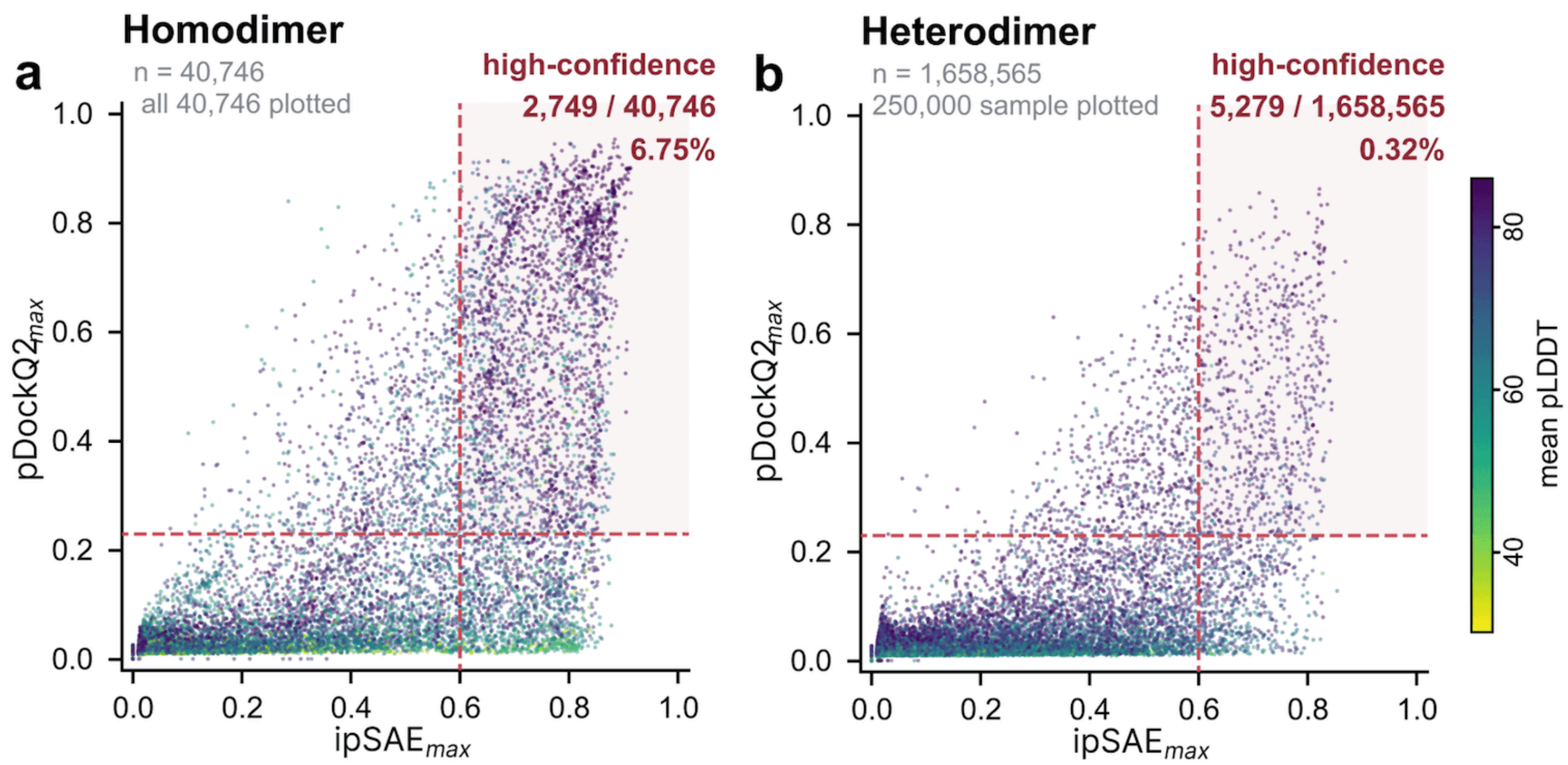

Supp. Fig 6. Homodimer & heterodimer production result. Predicted interface scores for every homodimer (a) and heterodimer (b) model, coloured by mean pLDDT. Dashed lines mark the two thresholds; the upper-right quadrant is the high-confidence set, with its count and rate given in place. Points are a random sample of at most 250,000 models per panel; all counts and rates are computed on the full population, not the sample.

bioRxiv preprint doi: https://doi.org/10.64898/2026.09.29.755321; this version posted October 1, 2026. The copyright holder for this preprint (which was not certified by peer review) is the author/funder, who has granted bioRxiv a license to display the preprint in perpetuity. It is made available under a CC-BY 4.0 International license.

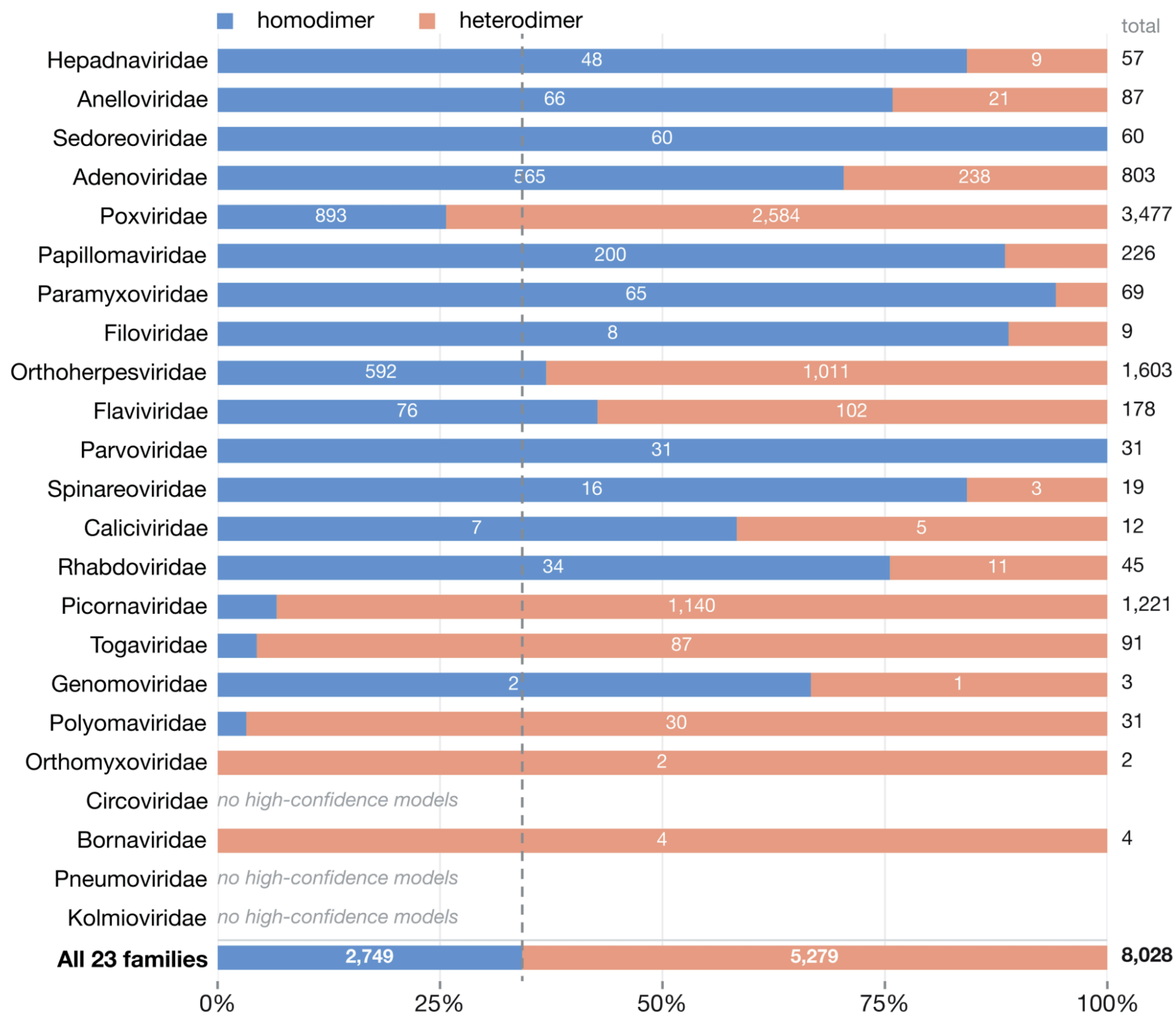

Supplementary Figure 7. Composition of the high-confidence predictions in each viral family. Each bar shows how one family's high-confidence models divide between pairs of a protein with a copy of itself and pairs of two different proteins of the same virus, scaled to 100%; the two counts are given inside the bar and the family total at the right. The dashed line marks all 23 families combined, shown in the bottom row. 3 families yielded no high-confidence model and so have no composition. Families are ordered as in **Fig. 3a**. Pairs of identical proteins make up 2.4% of the 1,699,311 predictions but 34% of the 8,028 high-confidence ones. The proportion differs widely between families and does not follow the ranking by success rate: *Poxviridae* contributes the most high-confidence models (3,477), of which 26% are identical pairs, while *Hepadnaviridae*, which has the highest success rate for identical pairs in **Fig. 3a**, contributes 57. Numbers of predictions attempted and success rates for every family are given in the accompanying table.

bioRxiv preprint doi: https://doi.org/10.64898/2026.09.29.755321; this version posted October 1, 2026. The copyright holder for this preprint (which was not certified by peer review) is the author/funder, who has granted bioRxiv a license to display the preprint in perpetuity. It is made available under a CC-BY 4.0 International license.

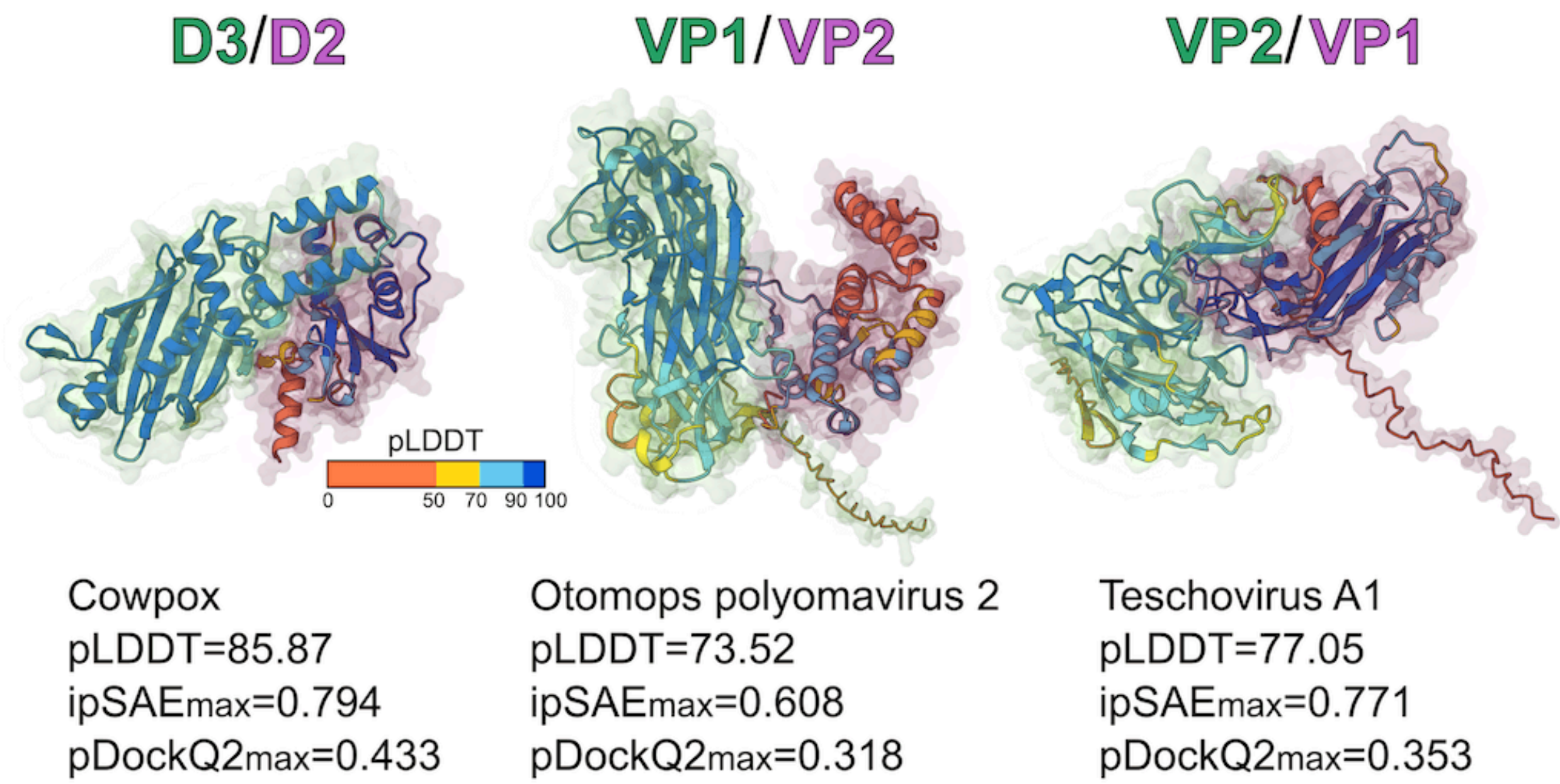

Supplementary Figure 8. Capsid assemblies.
Example high-confidence viral capsid protein heterodimers, from diverse viruses, that are otherwise absent or poorly represented on the PDB (from left to right: AF-0000000213303165, AF-0000000212018239, ,AF-0000000212074116). Ribbon diagrams are colour-coded by pLDDT prediction confidence, as denoted in the key, with transparent surface representations highlighting individual chains colour-coded by protein identity, as shown in the complex labels. Annotations provide average pLDDT values and interaction confidence metrics.

bioRxiv preprint doi: https://doi.org/10.64898/2026.09.29.755321; this version posted October 1, 2026. The copyright holder for this preprint (which was not certified by peer review) is the author/funder, who has granted bioRxiv a license to display the preprint in perpetuity. It is made available under a CC-BY 4.0 International license.

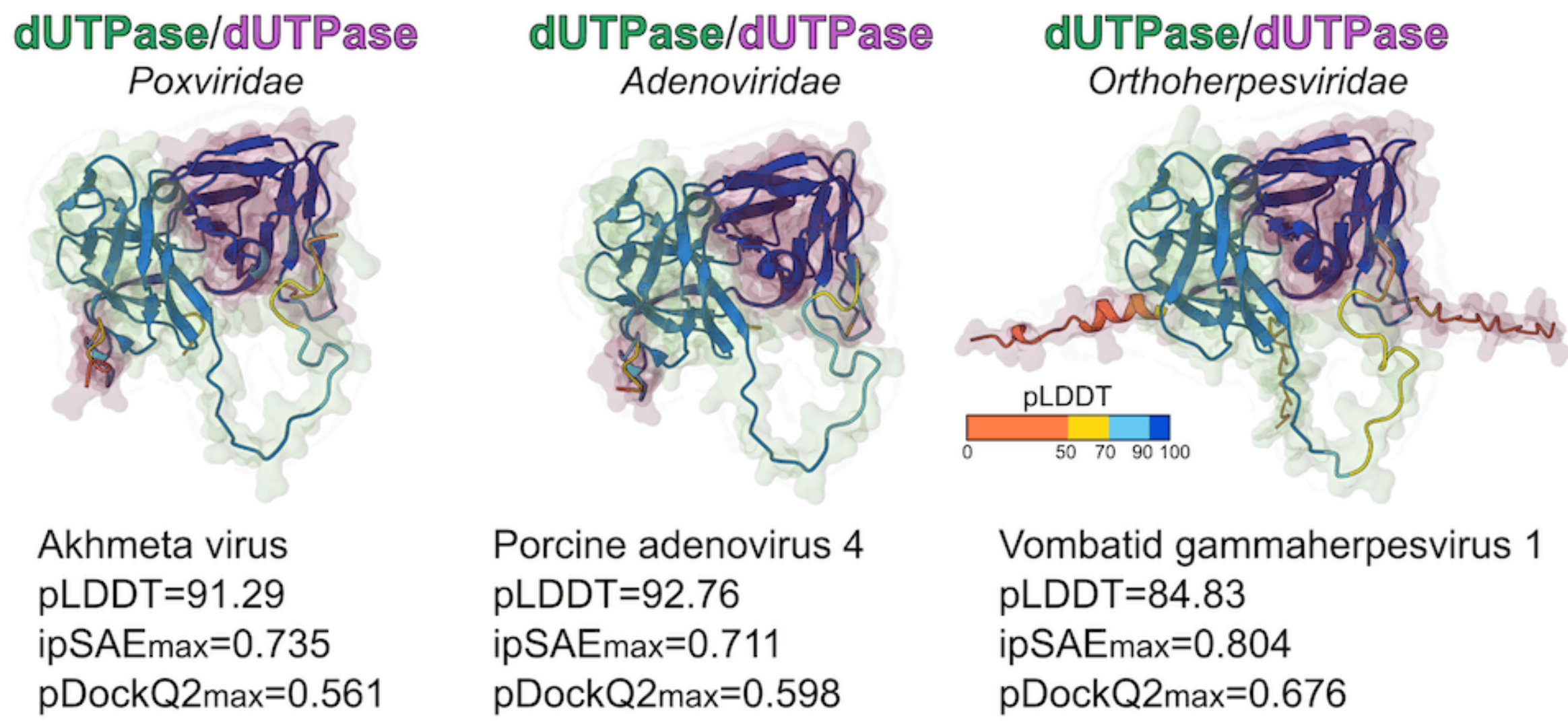

Supplementary Figure 9. A cross-family interface-cluster of viral dUTPase homodimers
Example structures from an interface-cluster of dUTPase homodimers, containing members from the *Poxviridae* (AF-0000000213202665), *Adenoviridae* (AF-0000000212119938), *Orthoherpesviridae* (AF-0000000212243866). Ribbon diagrams are colour-coded by pLDDT prediction confidence, as denoted in the key, with transparent surface representations highlighting individual chains colour-coded by protein identity, as shown in the complex labels. Annotations provide average pLDDT values and interaction confidence metrics.

bioRxiv preprint doi: https://doi.org/10.64898/2026.09.29.755321; this version posted October 1, 2026. The copyright holder for this preprint (which was not certified by peer review) is the author/funder, who has granted bioRxiv a license to display the preprint in perpetuity. It is made available under a CC-BY 4.0 International license.

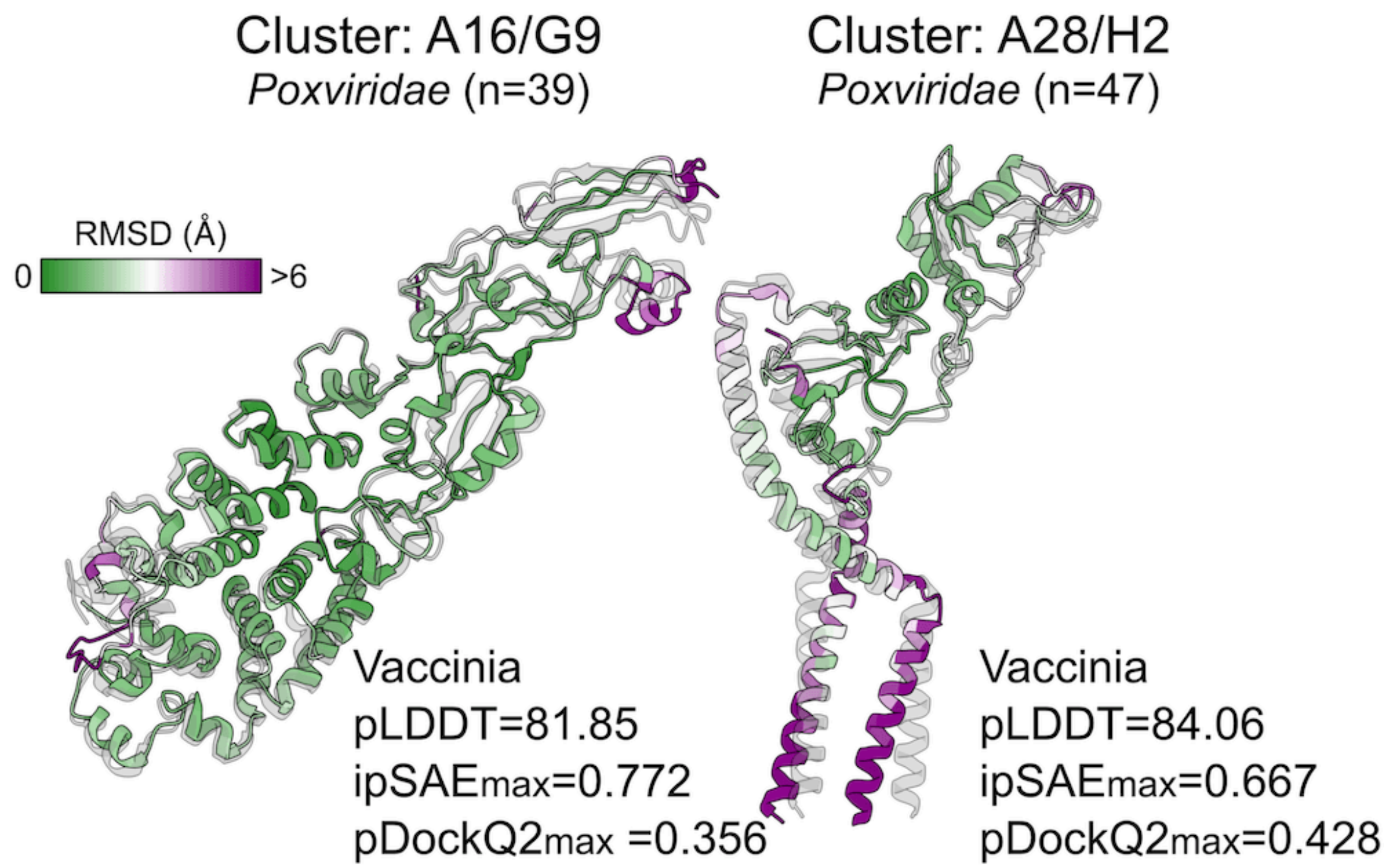

Supplementary Figure 10. Comparison of vaccinia EFC predictions with recent experimental structures
Dimer structures taken from two interface clusters representing components of the poxvirus entry fusion complex, A16/G9 and A28/H2, as in Fig. 4b. Ribbon diagrams are colour coded by C-α RMSD following superposition with recently published experimental structures, PDB references are shown as grey transparent ribbons (A16/G9 vs pdb_00009R0J, A28/H2 vs pdb_00009UZP). Regions of heterodimers that are not present in the experimental structures have been excluded from the visualisation. Annotations provide average pLDDT values and interaction confidence metrics. Cluster labels indicate complex identity, viral taxonomy and, in parentheses, number of members per cluster.

bioRxiv preprint doi: https://doi.org/10.64898/2026.09.29.755321; this version posted October 1, 2026. The copyright holder for this preprint (which was not certified by peer review) is the author/funder, who has granted bioRxiv a license to display the preprint in perpetuity. It is made available under a CC-BY 4.0 International license.

CATEGORY	PRODUCT NAMES	NAMES	CHAINS
Capsid	Capsid protein, Capsid protein C, Capsid protein VP0, Capsid protein VP1, Capsid protein VP2, Capsid protein VP3, Capsid protein VP4, Capsid protein VP60	8	2,028
Core / nucleocapsid	Core protein, Mature core protein	2	26
Other	3C like protease, 6K protein, Assembly protein E3, Assembly signal 2A, Cysteine protease NS2, E rns glycoprotein, Envelope glycoprotein E1, Envelope glycoprotein E2, Envelope protein E, Leader protease, Leader protein, N terminal protease, NS1, NS1 2, NS1 NS2, NS2, NS4, NTPase, Non structural protein 1, Non structural protein 2A, Non structural protein 3, Non structural protein 4A, Non structural protein 4B, Non structural protein 5A, Peptide 2k, Peptide pr, Protease 2A, Protease 3C, Protease NS2, Protease nsP2, Protease polymerase, Protein 2A, Protein 2A 1, Protein 2A 2, Protein 2A 3, Protein 2A 4, Protein 2A H NC, Protein 2A NTPase, Protein 2B, Protein 2C, Protein 3A, Protein 3B, Protein 3B 1, Protein 3B 2, Protein 3B 3, Protein PrM, Protein prM, RNA directed RNA polymerase, RNA directed RNA polymerase 3D POL, RNA directed RNA polymerase Methyltransferase NS5, RNA directed RNA polymerase nsP4, Serine protease Helicase NS3, Serine protease NS3, Serine protease helicase NS3, Serine protease subunit NS2B, Small envelope protein M, Spike glycoprotein E1, Spike glycoprotein E2, VPg, Viral genome linked protein, Viral protein genome linked, Viroporin p7, Viroporin p7 like, mRNA capping enzyme nsP1, p13, p6	66	5,483
Unclassified	Protein Y	1	20

Supplementary Table 1. Classification of polyprotein mature-product names.
All 77 distinct mature-product names in the dataset, grouped by the category assigned to them and together covering 7,557 chains. Products of a polyprotein are classified from the product name, because a UniProt keyword or Gene Ontology term attaches to the whole polyprotein and cannot indicate which product a chain is. Capsid and core are reported separately here and combined for compositional analyses; unclassified means the name does not identify the product. Criteria for proteins that are not polyprotein products are given in the accompanying table.
